# Association between thoracolumbar fascia injury and residual back pain following percutaneous vertebral augmentation: a systematic review and meta-analysis

**DOI:** 10.3389/fendo.2025.1532355

**Published:** 2025-04-22

**Authors:** Abdiaziz Ahmed Mohamed, Xu Xuyang, Zhang Zhiqiang, Jianghu Chen

**Affiliations:** ^1^ Department of Orthopedics, Northern Jiangsu People’s Hospital Affiliated Hospital to Yangzhou University, Yangzhou, Jiangsu, China; ^2^ Medical College of Yangzhou University, Yangzhou, Jiangsu, China; ^3^ Yangzhou Clinical Medical College of Xuzhou Medical University, Xuzhou, Jiangsu, China

**Keywords:** fascia injury, osteoporosis, compression fracture, meta-analysis, fragility fracture, residual back pain

## Abstract

**Objective:**

To evaluate the association between a thoracolumbar fascia injury (TLFI) and the development of residual back pain (RBP) following percutaneous vertebral augmentation (PVA).

**Background:**

Osteoporotic vertebral compression fractures (OVCF) commonly affect elderly individuals and those with osteoporosis, leading to pain and limited mobility. Percutaneous vertebral augmentation provides immediate pain relief and stabilization of the fractures. However, some patients experience residual pain after the treatment. Although recent studies have suggested a potential association, the role of TLFI in RBP remains inconclusive. The aim of this meta-analysis was to evaluate this association.

**Methods:**

A thorough search was performed across the PubMed, Medline, Embase, Web of Science, and Cochrane Library databases from inception to 31 December 2024 to identify studies examining the link between TLFI and RBP following PVA. A random-effects model was used to combine the outcome data to account for the potential heterogeneity among the included studies.

**Results:**

This meta-analysis included 13 studies with a total of 4,542 participants and a TLFI incidence rate of 28%. Univariate analysis indicated that patients with a TLFI were significantly more likely to develop RBP compared to those without a TLFI, with an odds ratio (OR) of 4.19 (95% CI: 2.49 to 7.05, I² = 76.9%). The sensitivity analysis identified two studies as significant influential outliers that contributed to the majority of the observed heterogeneity. Excluding these studies resulted in an OR of 4.62 (95% CI: 3.61 to 5.92, I² = 0%). The multivariate analysis confirmed a strong association between TLFI and RBP after adjusting for confounders and other risk factors, with an OR of 4.57 (95% CI: 3.28 to 6.37, I² = 81.5%). The sensitivity analysis identified three studies as significant influential outliers, and excluding them resulted in an OR of 4.79 (95% CI: 3.76 to 6.11, I² = 0%) with no heterogeneity. This finding further confirms the association with a more homogenous overall effect estimate.

**Conclusion:**

The pooled effect size of both univariate and multivariate analyses consistently demonstrated that a TLFI significantly increased the risk of developing RBP after PVA regardless of other related risk factors. Recognizing fascia injury as a potential source of postoperative pain in clinical practice could enhance the care of these patients and mitigate postoperative pain.

## 1 Introduction

Osteoporotic vertebral compression fractures (OVCF) are extremely common, particularly in elderly individuals and in those with osteoporosis (1, 2). Compression of the vertebrae causes these fractures, resulting in severe pain, limited mobility, and decreased quality of life (3, 4). OVCF is typically caused by low-energy trauma, although many patients do not report any traumatic incidents (5). However, the prevalence of osteoporosis and the occurrence of OVFs vary significantly across racial groups and geographic regions. These variations can be attributed to epigenetic and genetic factors. These factors not only influence bone mineral density but also uniquely influence and predispose different populations to fragility fractures (6). Percutaneous vertebral augmentation (PVA) is a minimally invasive treatment procedure for OVCFs that includes percutaneous vertebroplasty (PVP) and percutaneous kyphoplasty (PKP). These procedures have emerged as effective interventions that can alleviate back pain and stabilize vertebral fractures (7, 8). Percutaneous vertebroplasty involves the injection of bone cement into the fractured vertebra, stabilizing it and providing immediate pain relief (9). Percutaneous Kyphoplasty, on the other hand, involves the use of an inflated balloon to create a cavity within the vertebral body, with the objective of correcting deformity and restoring vertebral height prior to cement injection (10, 11). Despite the fact that PVA procedures provide immediate pain alleviation and improve the functionality of patients, moderate to severe postoperative pain may persist in certain individual patients (12, 13). According to the literature, a subset of OVCF patients, ranging from 5% to 32%, experienced residual back pain (RBP) following PVA procedures (14). Our findings also revealed similar results, the incidence of RBP was between 4.6% to 24.2% with an average of 13.9% across included studies in the analysis. It is essential to fully understand the root causes of residual back pain in these patients following vertebral augmentation procedures to maximize and revise treatment procedures, reduce the frequency of postoperative pain, and enhance long-term results for OVCF patients. Insufficient height restoration, cement leakage, inadequate cement distribution, advanced osteoporosis, sarcopenia, and intervertebral vacuum cleft are among the various risk factors identified in the literature as causes of residual back pain experienced by these patients following percutaneous vertebral augmentation procedures (15). The role of preoperative thoracolumbar fascia injury (TLFI) on residual back pain is still unknown and remains controversial and uncertain (16). Some studies have reported that a preoperative TLFI serves as a risk factor for back pain in the short term and is not a rare condition in OVCF patients but rather an overlooked condition, which becomes apparent after the pain associated with the fracture is alleviated (17, 18). The thoracolumbar fascia is a complex, multilayered connective tissue located in the lower back. It extends from the thoracic spine to the sacrum and plays an important role in the biomechanical stability and movements of the spine, such as forward flexion (19). Additionally, it provides attachment points to various paraspinal muscles, thereby facilitating the transmission of forces across the trunk and contributing to core stability (20). In patients with OVCFs, the presence of a TLFI is diagnosed by analyzing the signal produced by magnetic resonance imaging (MRI), which may manifest as posterior fascia edema or swelling. This meta-analysis aimed to evaluate the association between TLFI and residual back pain following PVA.

## 2 Methods

The present systematic review and meta-analysis were carried out following the principles outlined by the Preferred Reporting Items for Systematic Reviews and Meta-Analyses (PRISMA) (21) and the Cochrane Handbook for Systematic Reviews and Meta-analyses (22); however, this review was not registered in PROSPERO.

### 2.1 Literature search

We searched the PubMed, Medline, Embase, Web of Science, and Cochrane Library databases and conducted two separate searches: the first up to 31 January 2024 and a follow-up update search up to 31 December 2024 almost a year after the first search with the same search query used without change to ensure consistency. The following free search terms were used: osteoporotic vertebral compression fractures OR OVCF OR osteoporotic thoracolumbar compression fractures OR osteoporotic spinal compression fracture OR percutaneous vertebroplasty OR percutaneous kyphoplasty OR percutaneous cementoplasty OR percutaneous vertebral augmentation OR vertebral body augmentation OR percutaneous spinal augmentation OR vertebral augmentation OR Risk factor OR Predictor OR residual back pain OR residual low back pain OR persistent back pain OR chronic back pain OR recurrent pain OR thoracolumbar fascia injury. We used the Boolean operator AND to combine the title, abstract, and keyword phrases with medical subject headings (MeSH terms) to generate a broad search and identify the most relevant articles. The included studies were only published in English. Search terms were adjusted based on the database of interest. In addition, we performed a manual review of the references for eligible studies (see **Supplementary Data Sheet 1**).

### 2.2 Selection criteria

The study inclusion criteria followed the PICO guidelines and focused on patients diagnosed with osteoporotic vertebral compression fractures. The intervention or treatment option for this condition is percutaneous vertebral augmentation, involving either PVP or PKP. The control group was comprised of individuals who did not experience postoperative back pain. The outcome was the identification of TLFI as an independent risk factor for RBP along with its odds ratio in a multivariate analysis, which included observational studies or randomized controlled trials (RCTs) published in English as complete articles in peer-reviewed journals. Articles were excluded if they met the following criteria: (1) studies not including patients with OVCFs; (2) not reported TLFI as an independent risk factor in a multivariate analysis; (3) had missing or inadequate outcome data, such as multivariate analysis; (4) case reports, expert opinions, reviews, or commentaries/editorial letters; (5) abstracts only; (6) animal-related studies; and (7) non-English.

### 2.3 Search and selection

**Figure 1:**
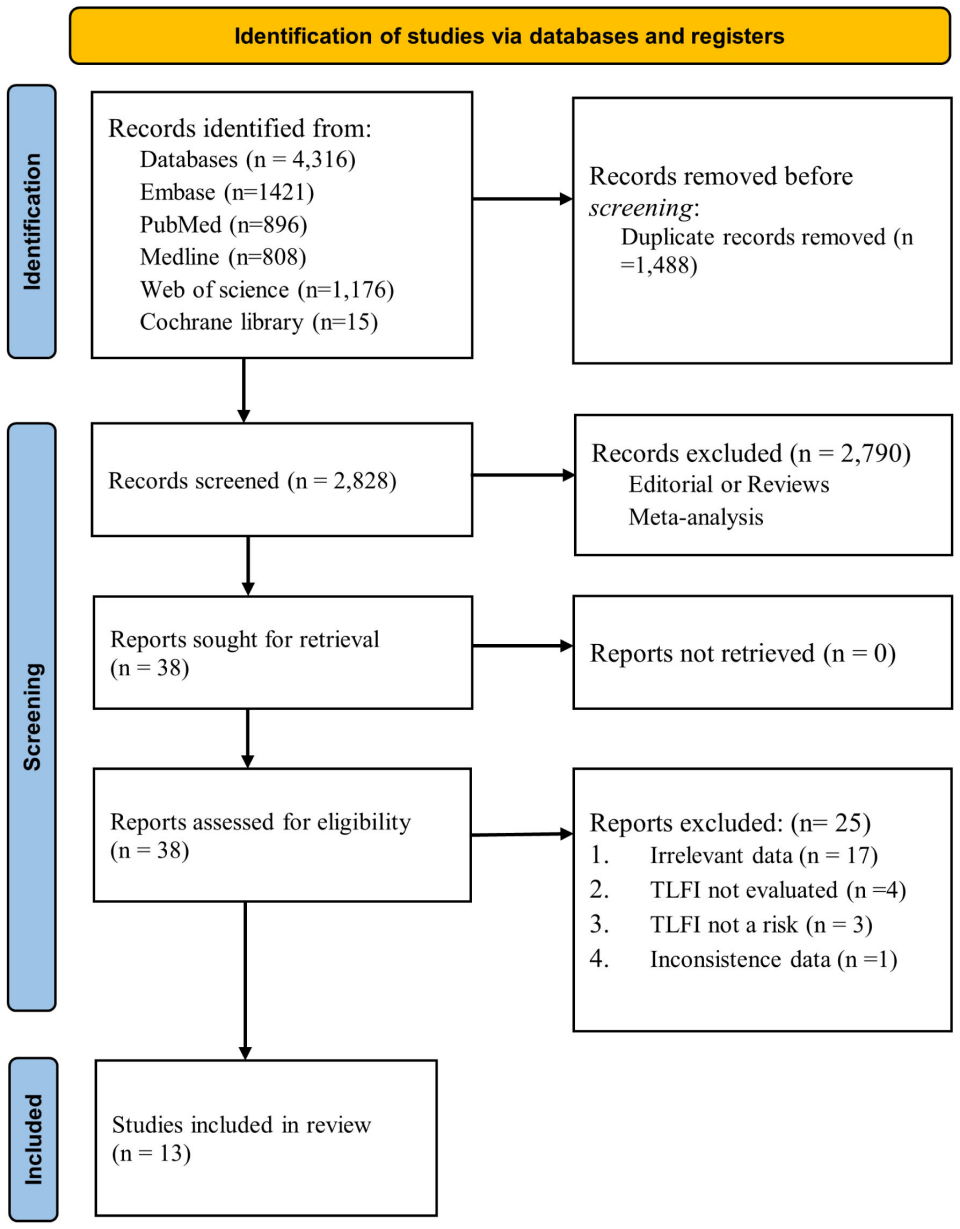
PRISMA flowchart for the literature search and selection process.

In total, 4,316 items were identified following a broad database search. After removing duplicates using EndNote v21 (28), 2,828 articles remained for screening. Two independent authors reviewed the titles. If the titles lacked sufficient information, we further examined the abstracts for inclusion, and 2,790 articles were excluded based on the inclusion and exclusion criteria. 38 articles were retrieved and assessed for analysis; subsequently, 25 were excluded due to explanations listed in **Figure 1**. The remaining 13 publications satisfied the study criteria for quantitative analysis in the meta-analysis. The primary outcome of our search was to assess whether TLFI was associated with RBP as an independent risk factor for OVCF following percutaneous vertebral augmentation.

### 2.4 Data extraction

Two authors completed the literature search and performed data extraction in accordance with the defined inclusion and exclusion criteria. They extracted and cross-verified the data using standardized data extraction forms. The author discussed and resolved instances of disagreement, seeking guidance from a third author as needed. General information of the studies collected was: (1) authors, year of publication, and study design; (2) patient characteristics, diagnosis, sample size, age, sex, and BMI; (3) intervention and control information; (4) incidence of TLFI (5); adjusted or matched variables (**Table 1**).

**Table 1:** Basic characteristics of the included studies.

References	Year	Country	Study design	Diagnosis	PVA procedure	Sample size (cases/Control)	Mean age (years)	Sex (Female %)	RBP (incidence rate%)	TLFI (incidence rate %)	Fellow-up period	Matched or adjusted variables
Yang et al. (23)	2019	China	Retrospective	OVCF	PVP	60/60	69.3	66.7	4.6%	50%	12 months	Age, sex, BMI
Li et al. (24)	2020	China	Retrospective	OVCF	PKP	52/163	75.4	66.5	24.19%	7.4%	1 month	Age, sex, BMI
Li et al. (25)	2021	China	Retrospective	OVCF	PVP	37/231	75	80	13.80%	6.7%	1 month	Age, sex, BMI, surgical level.
Ge et al. (26)	2022	China	Prospective	OVCF	PKP	81/731	70	77.4	11.08%	6.7%	1 month	Age, sex, surgical level
Gao et al. (27)	2023	China	Retrospective	OVCF	PVA	86/790	76.6	64.7	9.82%	44.7%	12 months	Age, sex, BMI, surgical level
Lin et al. (28)	2023	China	Retrospective	OVCF	PKP	47/234	74.5	82.6	16.95%	16.4%	6 months	Age, sex, BMI, surgical level
Wang et al. (29)	2023	China	Retrospective	OVCF	PVP	46/629	77	58.2	6.81%	28%	12 months	Age, sex, BMI, surgical levels
Wang et al. (30)	2023	China	Retrospective	OVCF	PKP	28/155	79	50%	15.30%	53%	12 months	Age, sex, surgical levels
Tu et al. (31)	2024	China	Retrospective	OVCF	PKP	46/221	71.5	82%	17.22%	19.1%	3 months	Age, sex, BMI, surgical level
Chen et al. (32)	2024	China	Retrospective	OVCF	PVP	17/143	75.3	81%	10.62%	23.75%	6 months	Age, sex, BMI
Zhang et al. (33)	2024	China	Retrospective	OVCF	PVP	34/114	75	77%	22.97%	66.89%	N/R	Sex, surgical level,
Shen et al. (34)	2024	China	Retrospective	OVCF	PKP	50/339	74.8	58%	12.85%	22.62%	2 days	Age, sex, BMI, surgical level
Chen et al. (35)	2024	China	Retrospective	OVCF	PVP	48/100	74.7	80%	8.0%	14.19%	1 month	Age, sex, BMI, surgical level

OVCF, Osteoporotic vertebral compression fractures; PVA, percutaneous vertebral augmentation; PVP, percutaneous vertebroplasty; PKP, percutaneous kyphoplasty; RBP, Residual back pain; TLFI, Thoracolumbar fascia injury; BMI, Body mass index.

### 2.5 Quality assessment

Since all the studies included in the analysis were observational studies, quality assessment was carried out using the Newcastle–Ottawa scale (36) (**Table 2**). Each study received a total of nine points based on quality, which was assessed across three broad classifications. There were four points for selection, two points for compatibility, and three points for outcome. A study quality score of six points or more was deemed to be good, whereas a score of five points or lower was regarded as low quality.

**Table 2:** Newcastle–Ottawa score for quality assessment.

Author	Selection				Comparability		Outcome			Overall score
	1	2	3	4	5	6	7	8	9	
Yang, 2019 (23)	*	*	*	*	*	**/**	*	*	*	8
Li, 2020 (24)	*	*	*	*	*	*	*	*	**/**	8
Li, 2021 (25)	*	*	*	*	*	*	*	*	**/**	8
Ge, 2022 (26)	*	*	*	*	*	**/**	*	*	**/**	7
Gao, 2023 (27)	*	*	*	*	*	*	*	*	*	9
Lin, 2023 (28)	*	*	*	*	*	**/**	*	*	**/**	7
Wang, 2023 (29)	*	*	*	*	*	**/**	*	**/**	*	7
Wang, 2023 (30)	*	*	*	*	*	*	*	*	*	9
Tu, 2024 (31)	*	*	*	*	*	*	*	/	/	7
Chen, 2024 (32)	*	*	*	*	*	/	*	*	*	8
Zhang, 2024 (33)	*	*	*	*	*	/	*	/	/	6
Shen, 2024 (34)	*	*	*	*	*	*	*	/	/	7
Chen, 2024 (35)	*	*	*	*	*	*	*	/	/	7

### 2.6 Statistical analysis

All data analyses were conducted using R software version 4.3.2 https://www.R-project.org/ (37). The analyses used the “meta”, “dmetar”, and “esc” packages (38–41) to determine the overall effect size for the outcome. The pooled effect sizes of the odds ratios (ORs) and 95% confidence intervals (CIs) were calculated. The extent of heterogeneity was assessed using Cochran’s Q test along with the I² statistic for the included publications. I² values ≥50% indicate heterogeneity and correspond to p < 0.05, as determined by Cochran’s Q test. If the observed I² value was ≥50, we investigated the possible reasons for heterogeneity and the studies contributing to it. Considering the prospect of variation across studies, a random-effects model was applied to combine the effect sizes. We used the DerSimonian–Laird estimator (42) to calculate the variance in study heterogeneity. We also applied Knapp–Hartung adjustments (43) to calculate the 95% CI of the overall pooled effect. To investigate the possible reasons and the studies contributing to heterogeneity, we adopted the leave-one-out analysis method (44). Statistical significance was set at P < 0.05.

#### 2.6.1 Sensitivity analysis

Influential and outlier study analyses were conducted to ensure the robustness of the overall pooled effect estimates and to investigate each study’s contribution to both observed heterogeneity and overall effect size. Outliers were identified using the “find.outliers” function in the “dmetar” package in the univariate analysis with two studies (31), and (34), identified as outliers. Similarly, in the multivariate analysis, one study (30) was also recognized as an outlier. Furthermore, we carried out an influence diagnostic analysis to identify influential studies that may distort the pooled effect estimate in one direction or another using the “influenceAanalysis” function in the same package. The influence diagnostic analysis included multiple diagnostic tests, namely, the Baujat plot (45), influence diagnostic analysis according to Viechtbauer and Cheung (44), leave-one-out analysis, and graphical display of heterogeneity (GOSH) plot (46) diagnostics.

#### 2.6.2 Baujat diagnostic plot

**Figure 2:**
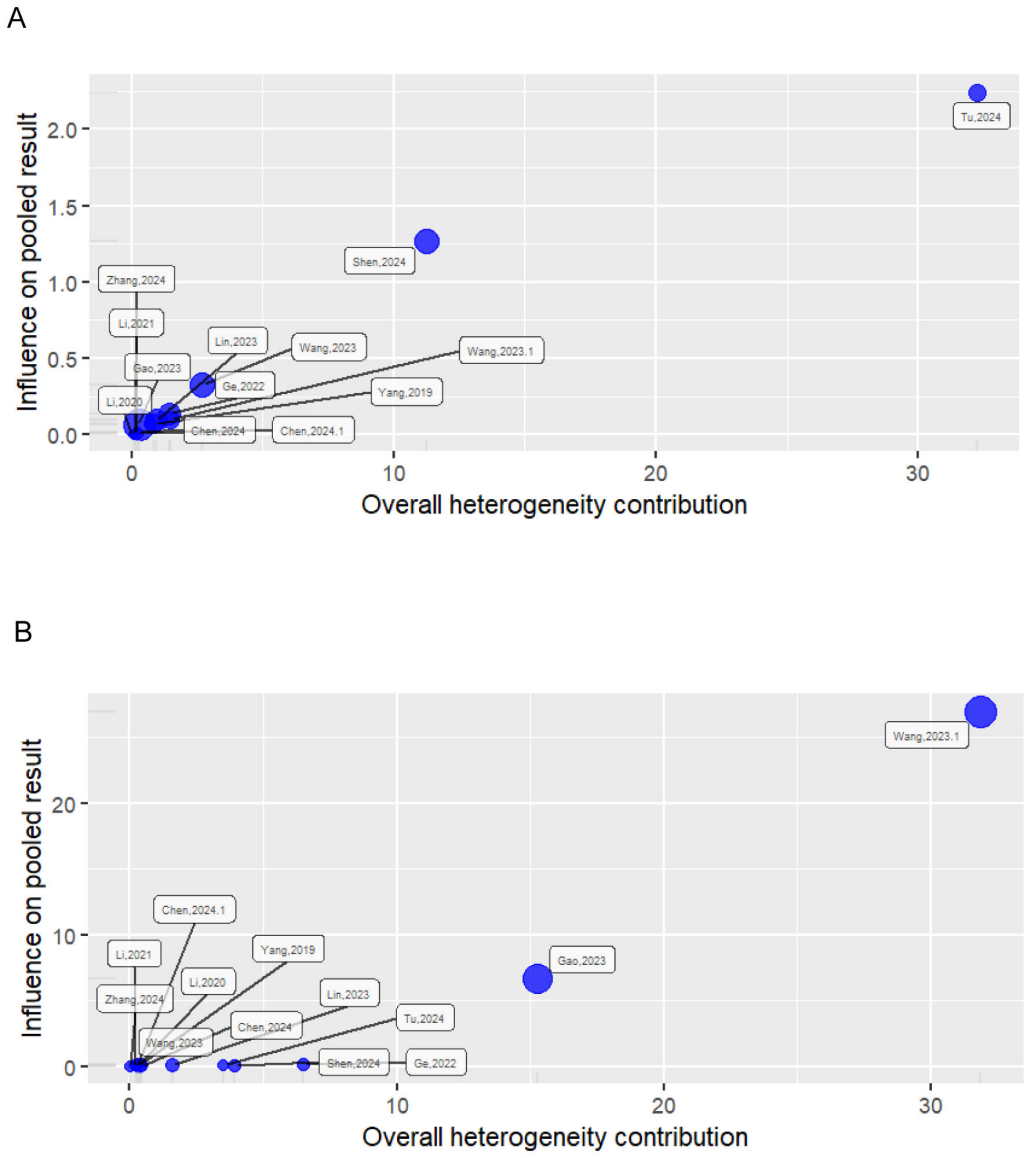
Baujat plot showing the influence of studies on heterogeneity and pooled effect. **(A)** Univariate analysis; **(B)** multivariate analysis.

The Baujat plot detects how each study contributes to heterogeneity based on Cochran’s Q and how they influence the overall effect using the leave-one-out method (45). Studies concentrated on the right side of the plot heavily contributed to the heterogeneity of the meta-analysis. Studies on the upper right side are considered particularly influential and contribute to both heterogeneity and overall effect size. In both the univariate and multivariate analyses, two studies were found to be influential outliers, namely (31) and (34) and (27) and (30), respectively, and these excessively contributed to both heterogeneity and the overall effect estimate (**Figure 2**).

#### 2.6.3 Influence diagnostics

**Figure 3:**
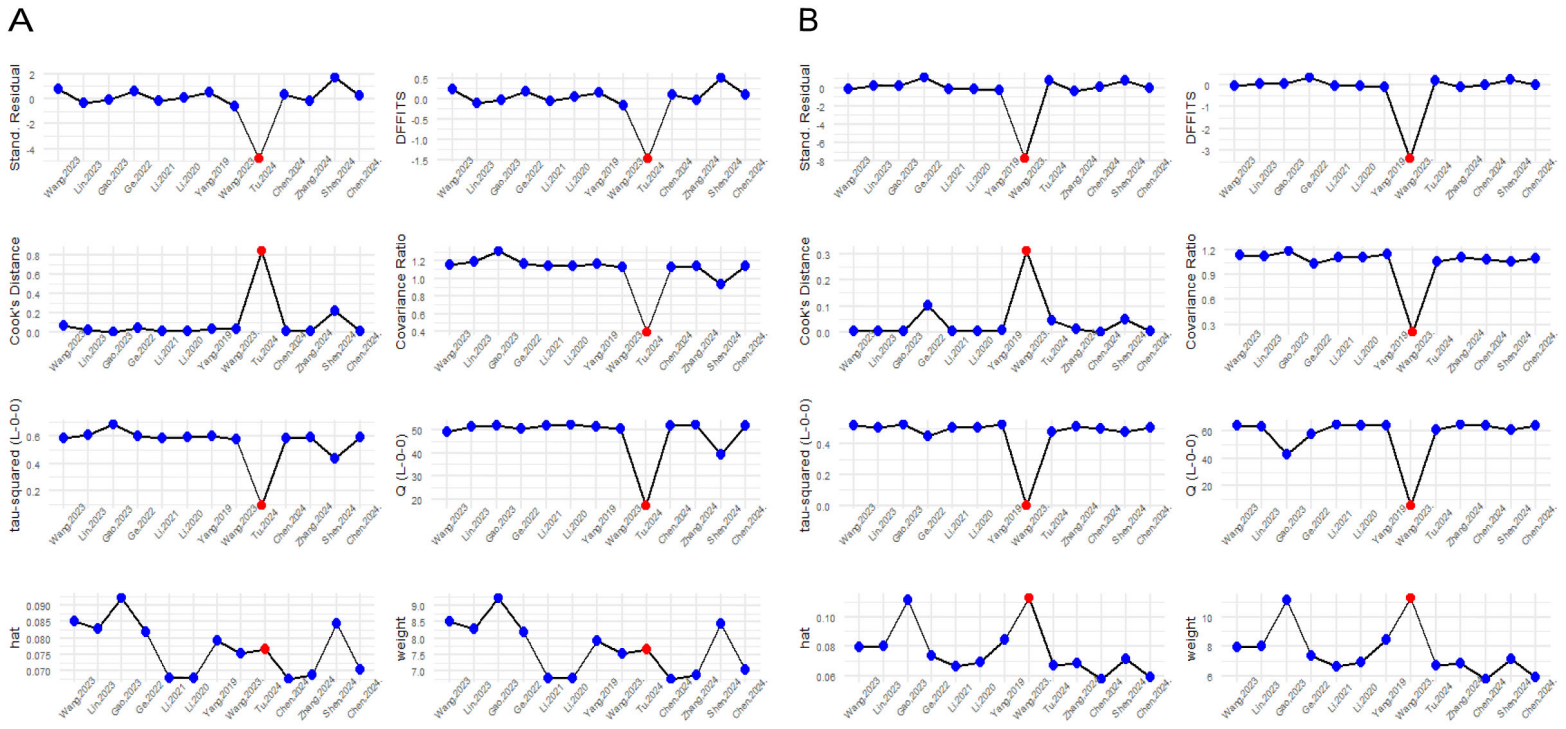
Influence diagnostic plot showing different influence diagnostic metrics. **(A)** Univariate analysis; **(B)** Multivariate analysis.

Influence diagnostics display and detect each study’s influence on the overall effect size of the meta-analysis (44). This influence diagnostic displays different plots that measure different influence diagnostic metrics. Studies with extreme values that may distort the overall effect estimate are shown in red in the different plots. This diagnostic test flagged the same studies as the previous sensitivity analysis in both the univariate and multivariate analyses, namely (31) and (34) and (27) and (30), respectively, as shown in **Figure 3**.

#### 2.6.4 Leave-one-out-analysis method

**Figure 4:**
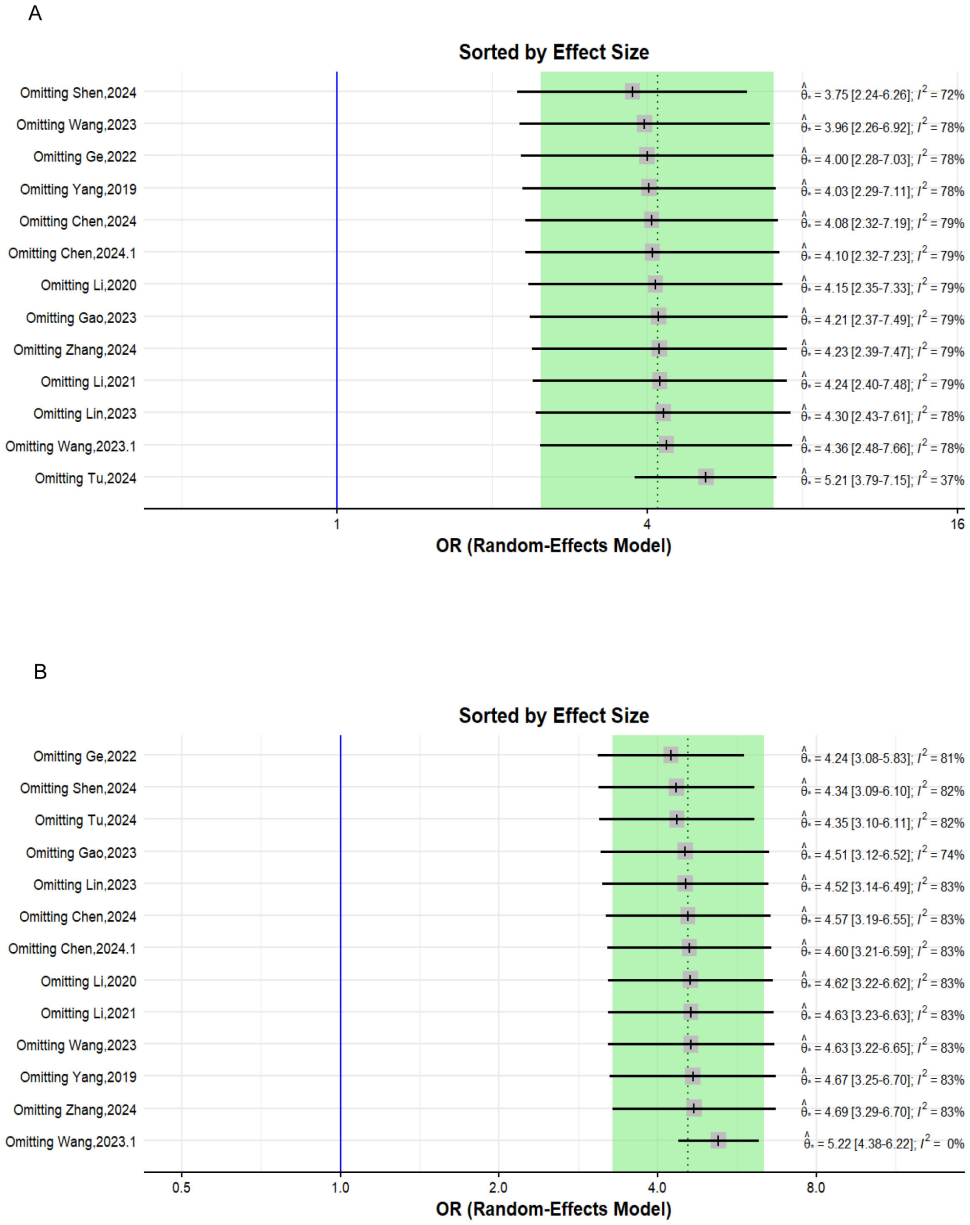
Forest plot depicting the leave-one-out sensitivity analysis. **(A)** Univariate analysis; **(B)** multivariate analysis.

The leave-one-out method sensitivity analysis omits one study at a time and recalculates the overall pooled effect size each time. Determining how both heterogeneity and the overall effect estimate change as different studies are excluded each time. In our analysis, the lowest heterogeneity and most robust overall effect estimate were reached after excluding the studies previously flagged by the other sensitivity analyses in both the univariate and multivariate analyses (**Figure 4**).

#### 2.6.5 GOSH plot diagnostics

**Figure 5:**
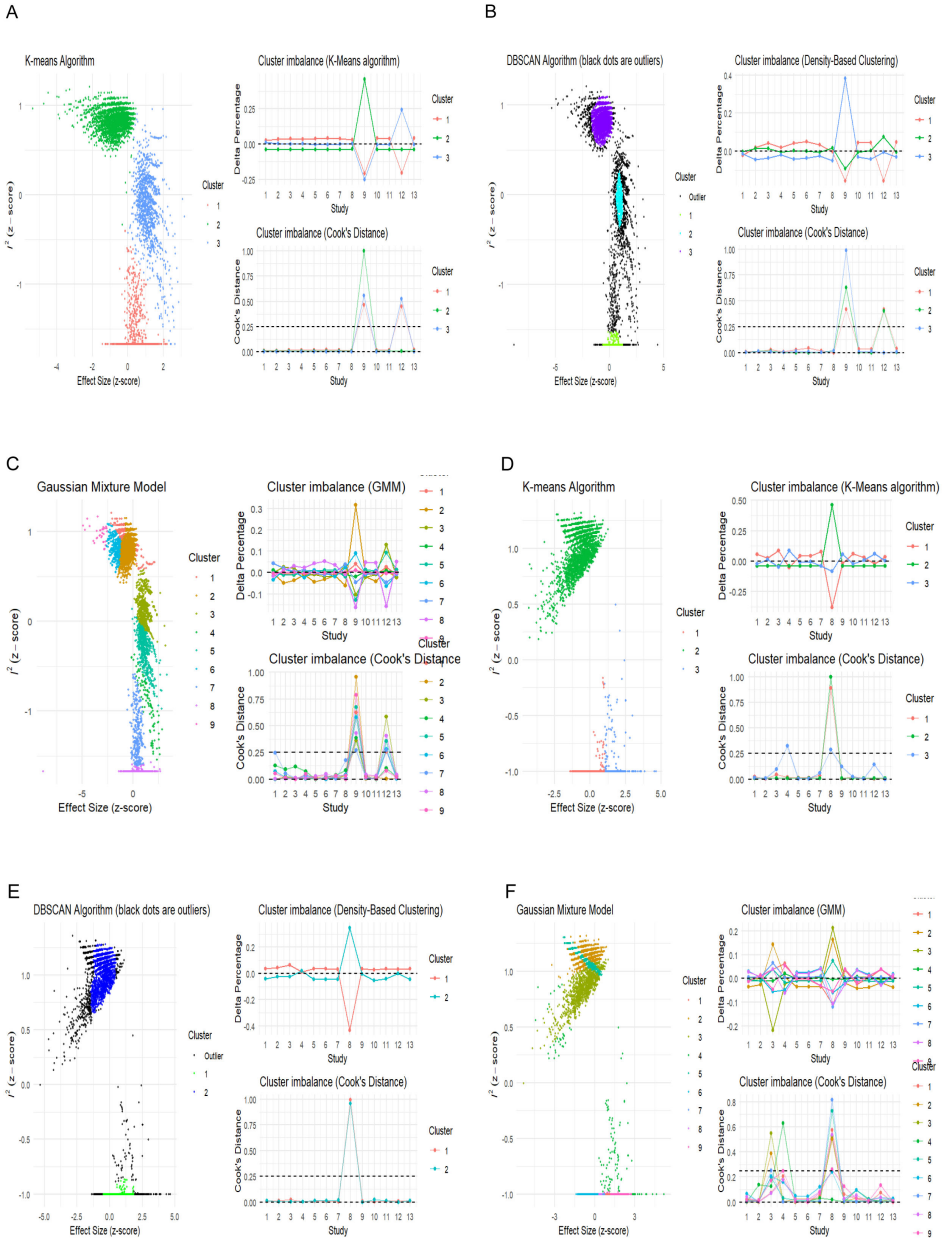
Unsupervised machine learning algorithms detecting influential studies. **(A–C)** Univariate analysis; **(D–F)** multivariate analysis.

GOSH plots (46) are another method that explores the pattern of heterogeneity in a meta-analysis by fitting all possible subsets of the studies into clusters to detect which study combinations contribute to heterogeneity. This can be achieved using the “Gosh.Diagnostics” function in the “dmetar’ package that uses three clustering or unsupervised algorithm K-means clustering (47), density reachability and connectivity clustering (DBSCAN) (48), and Gaussian mixture models (49) to display the heterogeneity pattern and study combinations that most likely contribute to it. In our meta-analysis, in both the univariate and multivariate analyses, the GOSH plot demonstrated an apparently high heterogeneity-effect estimate combination pattern (**Supplementary Figure 2**). This indicates that more than one study contributed to the observed heterogeneity of the effect size. The results of both the univariate and multivariate analyses of the different clustering algorithms are shown in **Figure 5**.

**Figure 6:**
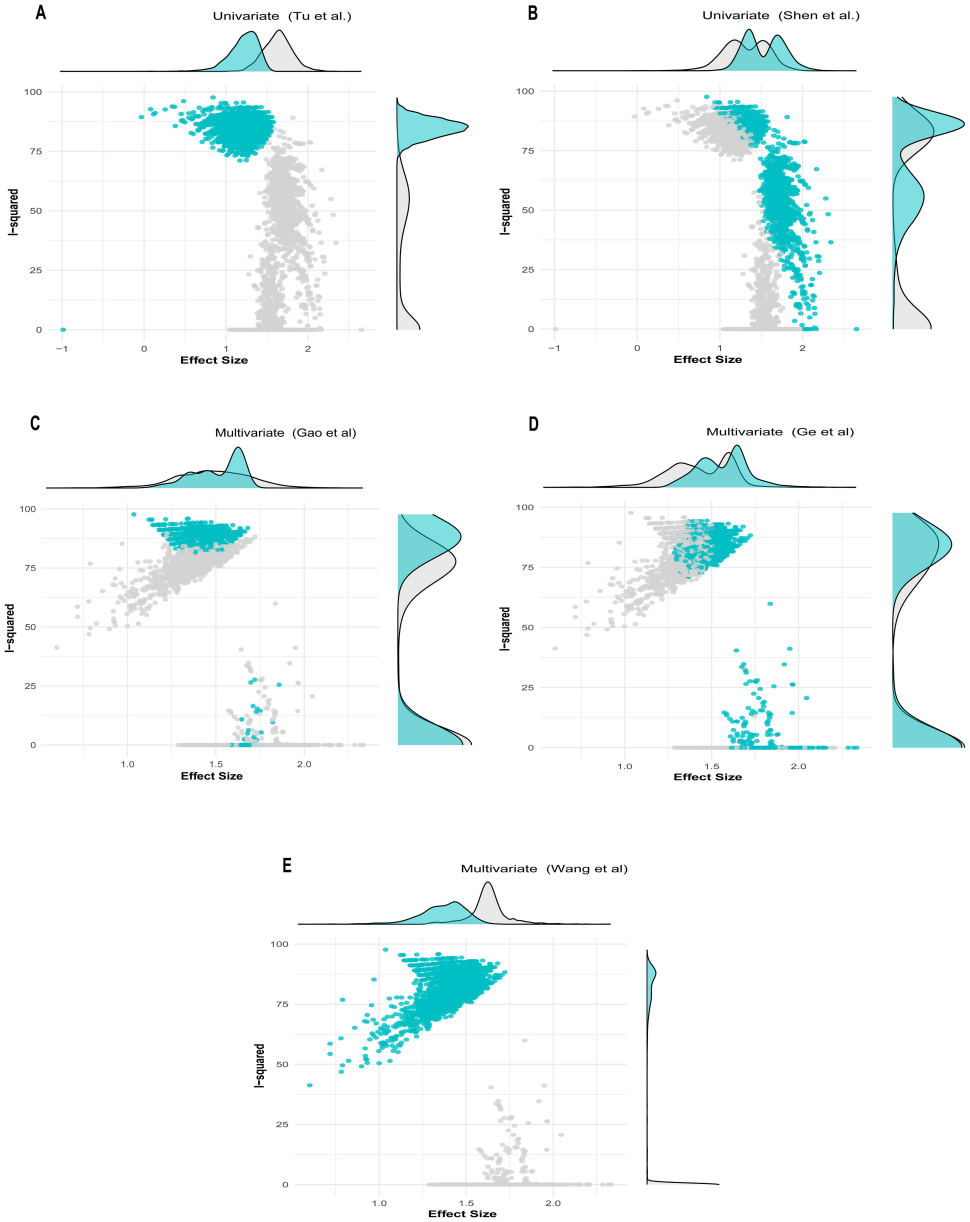
GOSH plot showing influential studies with shaded points depicting when the influential study is included in the analysis. **(A, B)** Univariate analysis; **(C–E)** multivariate analysis.

The univariate GOSH plot clustering algorithm results in identifying potential outliers contributing to heterogeneity are as follows (**Figure 6**):

- K-means: Study 9 (31) and Study 12 (34).
- DBSCAN: Study 9 (31) and Study 12 (34).
- Gaussian mixture model: Study 9 (31) and Study 12 (34)

In the univariate analysis, all cluster combinations incorporating the study by Tu et al. (31), exhibited high heterogeneity with low effect size, indicating the influential nature of this study (**Figure 6A**). Similarly, all the results in which the study by Shen et al. (34) was included demonstrated reduced heterogeneity contribution but high effect size, rendering this study influential due to its substantial effect on underestimating the overall effect size (**Figure 6B**).

The multivariate GOSH plot clustering algorithm results in identifying potential outliers contributing to heterogeneity are as follows (**Figure 6**):

- K-means: Study 8 (30) and Study 4 (26).
- DBSCAN: Study 8 (30).
- Gaussian mixture model: Study 4, Study 8, and Study 3 (26, 27, 30)

In the multivariate analysis, the results from the combinations of studies in which (26) and (27) were included exhibited a small degree of heterogeneity contribution. However, the study by Gao et al. (27), due to its narrow confidence interval, received a high weight and was recognized as an influential study despite its average effect size (**Figure 6C**). Conversely, the results incorporating the study by Ge et al. (26) demonstrated comparable heterogeneity contribution to that of (27). However, due to its substantial effect contribution relative to the overall effect size, it was considered influential. (**Figure 6D**). Similarly, the clusters that included the study by Wang et al. (30) demonstrated that this study contributed the highest level of heterogeneity. Despite having the smallest effect size among the studies included in the analysis, this study was previously identified as an influential outlier. (**Figure 6E**).

## 3 Results

### 3.1 Basic characteristics of the included studies

The evaluation included 13 observational studies, consisting of 12 retrospective cohort studies and one prospective research study. These studies collectively involved a study population of 4,542 individuals. Other important parameters from the included studies are listed in the baseline characteristics in **Table 1**. All studies assessed the association between TLFI as a risk factor and RBP, among other risk factors, following percutaneous vertebral augmentation.

### 3.2 Findings of the included studies

Yang et al. (23) conducted retrospective case-control research to identify the risk factors for persistent back pain after PVP. From 1,316 patients who underwent PVP for OVCF, 120 were selected. In total, 60 patients reported residual back pain (VAS score >4) 1 month postoperatively and were compared with 60 patients who did not report residual back pain. Univariate regression analysis revealed that the prevalence of TLFI was 71.7% in the case group and 28.3% in the control group, suggesting that TLFI is a potential risk factor for RBP. Multiple logistic regression analysis adjusted for confounders confirmed TLFI as an independent risk factor associated with RBP post-PVP (OR = 3.805; P = 0.002). Li et al. (24) performed a retrospective study to identify risk factors for residual back pain after PKP in 809 patients with osteoporotic vertebral compression fractures. The final analysis included 215 patients: 52 with moderate-to-severe residual pain (VAS ≥4) 1 month postoperatively and 163 with no or mild pain as controls. Univariate analysis showed that TLFI incidence was 17.3% in the case group and 4.3% in the control group. Multivariate logistic regression adjusted for other risk factors indicated that TLFI was independently associated with residual back pain post-PKP (OR = 4.11; P = 0.014). Li et al. (25) conducted a retrospective analysis to identify risk factors for persistent back pain after PVP. The study included 268 patients with OVCFs divided into residual pain (VAS score ≥4 after 1 month, n=37) and non-residual pain groups (n=231). They observed a TLFI incidence of 16.2% in the residual pain group and 5.2% in the non-residual pain group. Multiple logistic regression analysis showed that TLFI was an independent risk factor for residual pain post-PVP (OR, 3.965; P = 0.022). Similarly, Ge et al. (26) reported that in a population of 731 patients who underwent percutaneous kyphoplasty, 81 developed residual back pain after analyzing the risk factors in a prediction model. In a univariate analysis, they found that TLFI was associated with residual back after PKP surgery (OR, 6.933; P=<0.001) and the multivariate analysis indicated it was an independent risk factor for postoperative pain (OR, 11.377; p <0.001). Gao et al. (27) retrospectively reviewed the data of individuals treated with PVA, both percutaneous vertebroplasty and percutaneous kyphoplasty, to assess the causes of residual pain following the operation. In total, 86 patients were classified in the residual back pain group based on a VAS score ≥4, and 790 patients were in the control group. In a univariate analysis, they found that posterior fascia injury was associated with postoperative back pain with an incidence of 73% in the RBP group compared to 41.6% in the control group. In a multivariate analysis, they found that fascia injury was an independent risk factor associated with residual back pain (OR,5.23; P=<0.001). Lin et al. (28) analyzed retrospective data to identify the risk factors for residual back pain and developed a predictive nomogram after percutaneous kyphoplasty. They categorized subjects into a residual back pain group with a VAS score ≥4 1 month postoperatively and a non-residual back pain group, with 47 patients in the RBP group and 234 in the control group. Univariate analysis revealed that TLFI was associated with persistent pain in 31.9% of cases versus 13.2% of controls. Multivariate logistic regression, after accounting for confounders, indicated that TLFI was independently associated with residual back pain (OR, 5.36; P < 0.001). Wang et al. (29) investigated the risk factors associated with RBP following percutaneous vertebroplasty. In a study of 675 patients with OVCFs, 46 developed RBP (VAS score ≥4) 1 month postoperatively. The univariate logistic regression analysis showed that TLFI was present in 71.7% of the RBP cases compared to 24.8% of the control group. The multivariate analysis adjusted for other risk factors confirmed TLFI as a significant independent risk factor for RBP (OR, 4.083; P= 0.032). Wang et al. (30) conducted a risk factor analysis in a retrospective study on the causes of postoperative pain following PKP in patients with OVCFs. They divided 183 patients who received PKP into RBP and control groups based on a VAS score ≥4 postoperatively, although a pain measurement cutoff was not reported. A TLFI was diagnosed using preoperative MRI fat-suppression sequences. In univariate analysis, TLFI was present in 71.4% of the cases and 49.7% of the control group. After adjusting for confounders in the multivariate logistic regression, TLFI was identified as an independent risk factor for back pain (OR, 1.528; P<0.001). Tu et al. (31) evaluated risk factors for residual pain following PKP and developed a risk prediction model using data from 267 patients with OVCFs. RBP was defined as a VAS score ≥4 1 day postoperatively, dividing patients into RBP and non-RBP groups. A TLFI was identified based on preoperative MRI signal changes, low signal on T1-weighted images (T1WI), and high signal on both T2-weighted images (T2WI) and Short-TI Inversion Recovery (STIR). Multivariate logistic regression analysis, controlling for other factors, revealed that TLFI was independently associated with RBP (OR, 9.1; P < 0.01). Chen et al. (32) investigated the impact of enhanced central sensitization on RPB and its connection to RBP after a PVP procedure and related risk factors. RBP was defined as a VAS score of ≥4 at 1 d, 2 weeks, and 1 month after PVA. In the multivariate logistic regression analysis, TLFI was recognized as an independent risk factor for RBP and was defined according to preoperative MRI signal changes, low signal on T1W1, and high signal intensity on T2W1 and STIR sequences. Zhang et al. (33) concluded in a multivariate logistic regression analysis that preoperative TLFI is an independent risk factor for RPB post-PVA interventions. However, this study did not report a specific VAS score cut-off point or TLFI diagnostic method but referenced TLFI findings from previous studies (29). Shen et al. (34) examined the short-term risk factors associated with RBP after PKP. A VAS score of ≥4 at 2 days postoperatively was defined as RBP. A TLFI was defined as the presence of preoperative fascia injury and was diagnosed using MRI signal intensity changes, low signal on T1W1, and high signal on both T2W1 and fat-suppressed sequences. The multivariate risk analysis showed that preoperative TLFI was an independent risk factor for RBP after PKP surgery. Chen et al. (35) likewise examined the risk factors associated with RBP in patients who underwent PVP. The presence or absence of RBP was defined as a VAS score ≥4 immediately and 1 month postoperatively. The TLFI diagnostic method was not explicitly reported but referenced (28, 31). After adjusting for related risk factors in the multivariate logistic regression analysis, the presence of a preoperative TLFI was a risk factor that could lead to postoperative pain.

### 3.3 Results of current meta-analysis

**Figure 7:**
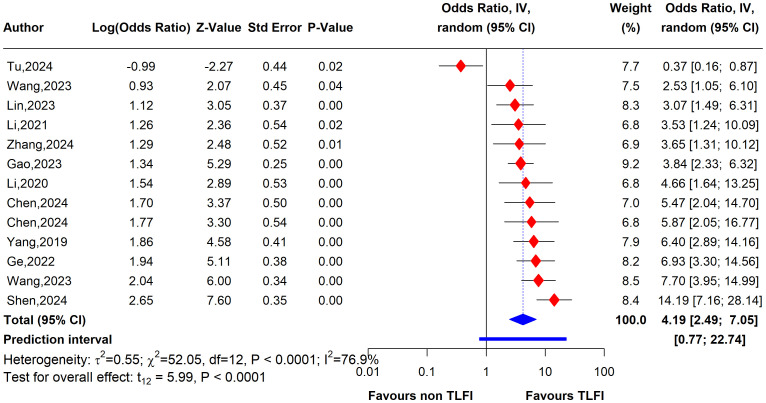
Forest plot depicting an association between TLFI and RBP following PVA with influential studies and outliers included (univariate analysis).

**Figure 8:**
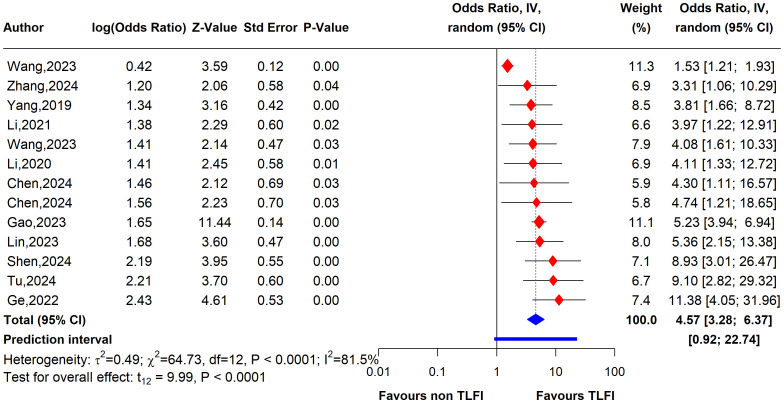
Forest plot depicting an association between TLFI and RBP following PVA with influential studies and outliers included (multivariate analysis).

The univariate analysis revealed that patients with a TLFI were significantly more likely to develop RBP than those without a TLFI. The pooled results of 13 studies indicate that the odds ratio (OR) for developing RBP in TLFI patients is 4.19 (95% CI: 2.49 to 7.05, I² = 76.9%), with the presence high level of heterogeneity. This suggests a more than three-fold increase in risk following PVA, as shown in **Figure 7**. The multivariate analysis of the pooled effect also demonstrated a substantial correlation between TLFI and the risk of RBP development post-PVA after accounting for confounders and other risk factors related to RBP, with an OR of 4.57 (95% CI: 3.28 to 6.37, I² = 81.5%) (**Figure 8**). However, high heterogeneity was observed in the multivariate analysis, indicating a considerable difference between the studies.

#### 3.3.1 Univariate sensitivity analysis

**Figure 9:**
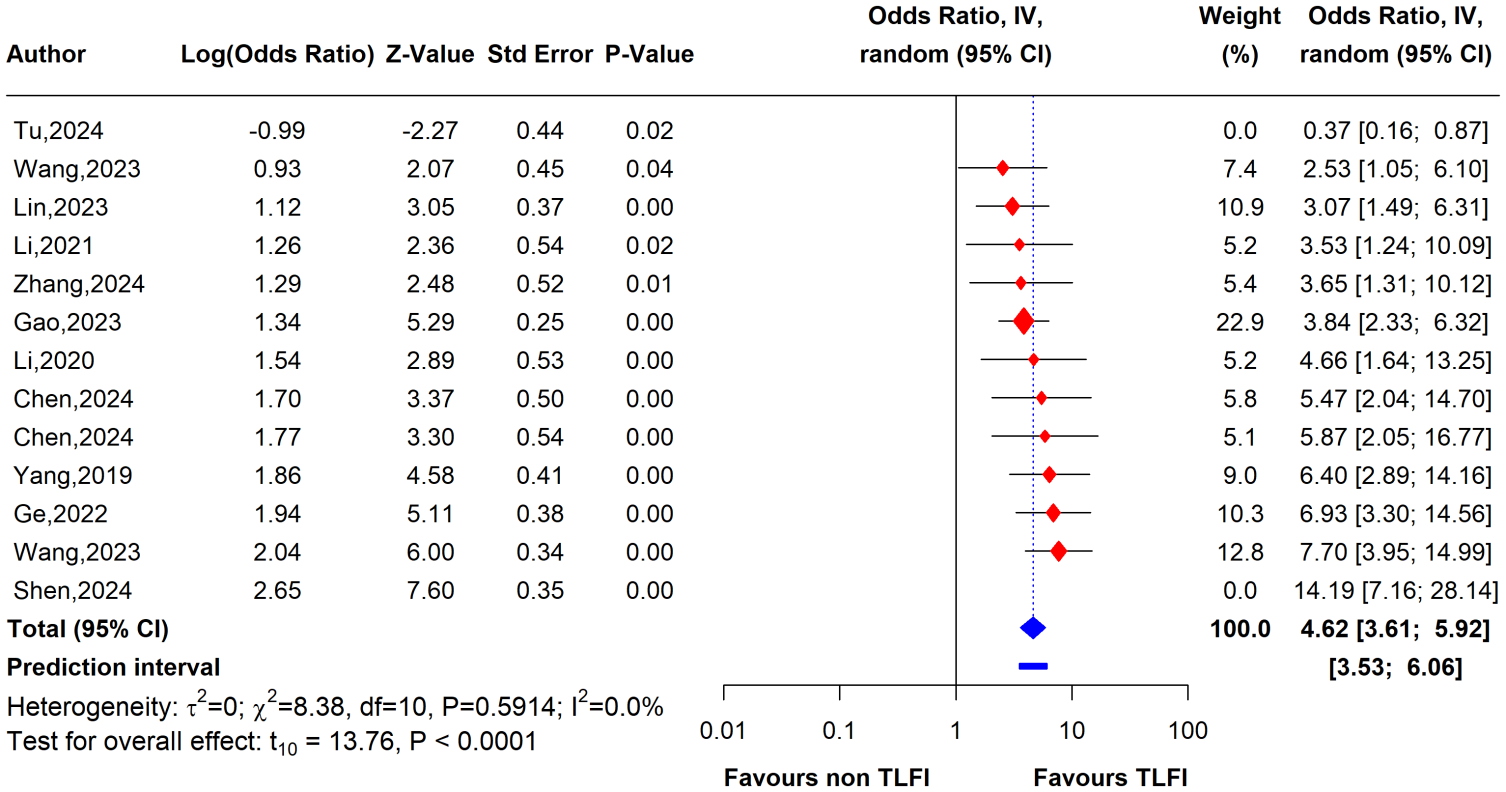
Forest plot depicting an association between TLFI and RBP following PVA with influential studies and outliers excluded (univariate analysis).

To evaluate the heterogeneity of these results and assess the influential studies contributing to it, a sensitivity analysis was performed and all different sensitivity analysis metrics identified two studies, i.e., (31) and (34) as influential outliers contributing to the majority of the observed heterogeneity and underestimating the overall effect size. After excluding these two studies, the overall effect size increased to an OR of 4.62 (95% CI: 3.61 to 5.92, I² = 0%) with no heterogeneity, indicating a more homogenous and stable estimate and reinforcing the significant association between TLFI and RBP (**Figure 9**).

#### 3.3.2 Multivariate sensitivity analysis

**Figure 10:**
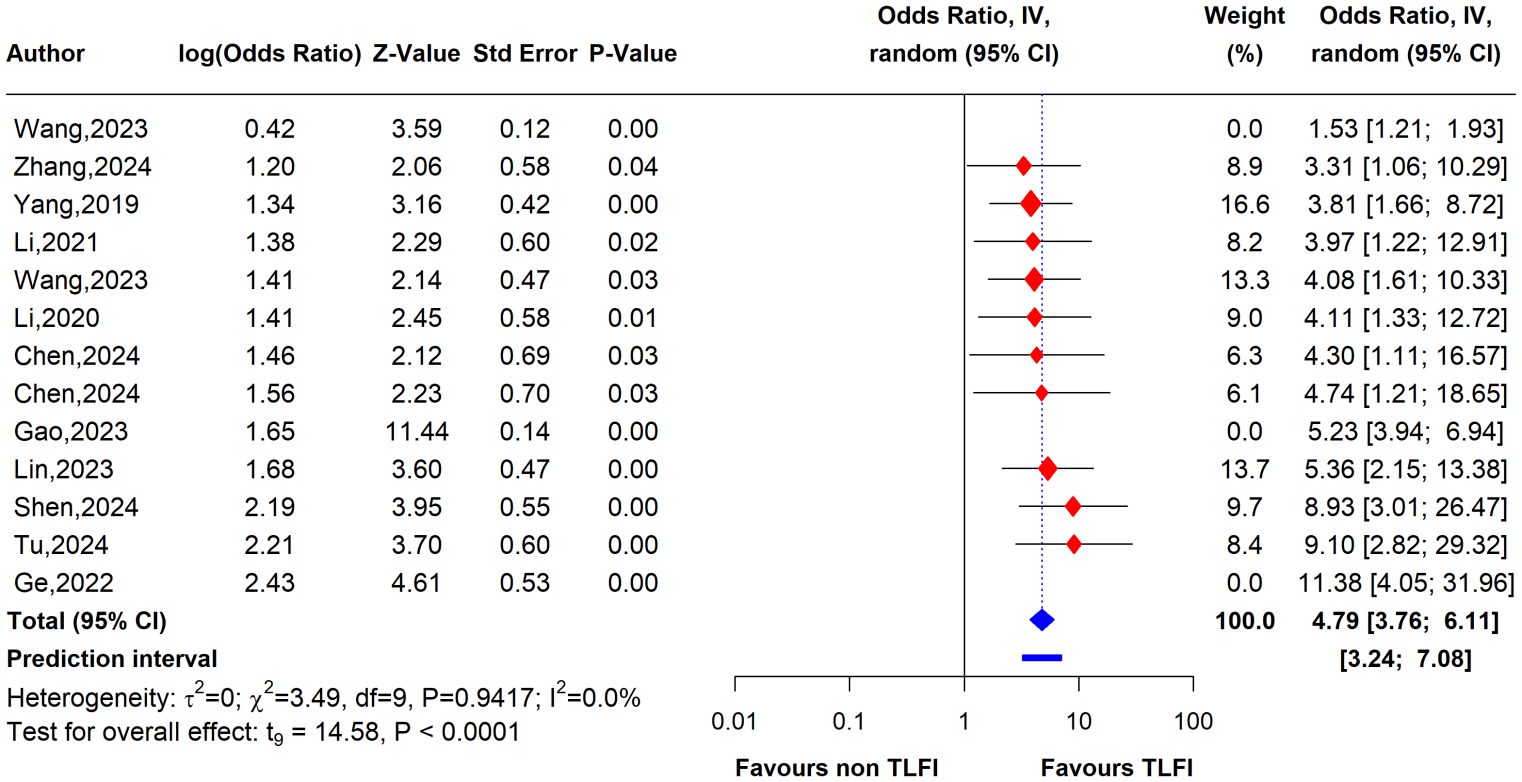
Forest plot depicting an association between TLFI and RBP following PVA with influential studies and outliers excluded (multivariate analysis).

To assess the heterogeneity of these results and investigate the influential studies contributing to it, a sensitivity analysis using the leave-one-out method was performed. The sensitivity analysis identified three studies, i.e., (26, 27, 30), to be influential and outliers contributed significantly to the high heterogeneity observed and overall effect size. After excluding these studies, the overall effect size was recalculated, showing a somewhat stronger and more homogenous estimate than the initial one with an OR of 4.79 (95% CI: 3.76 to 6.11, I² = 0%), with no heterogeneity. This indicates that the initial results were affected by the presence of influential and outlier studies that underestimated the overall effect size. However, the recalculated effect size confirmed a significant association between TLFI and increased risk of RBP following PVA (**Figure 10**)

### 3.4 Publication bias

**Figure 11:**
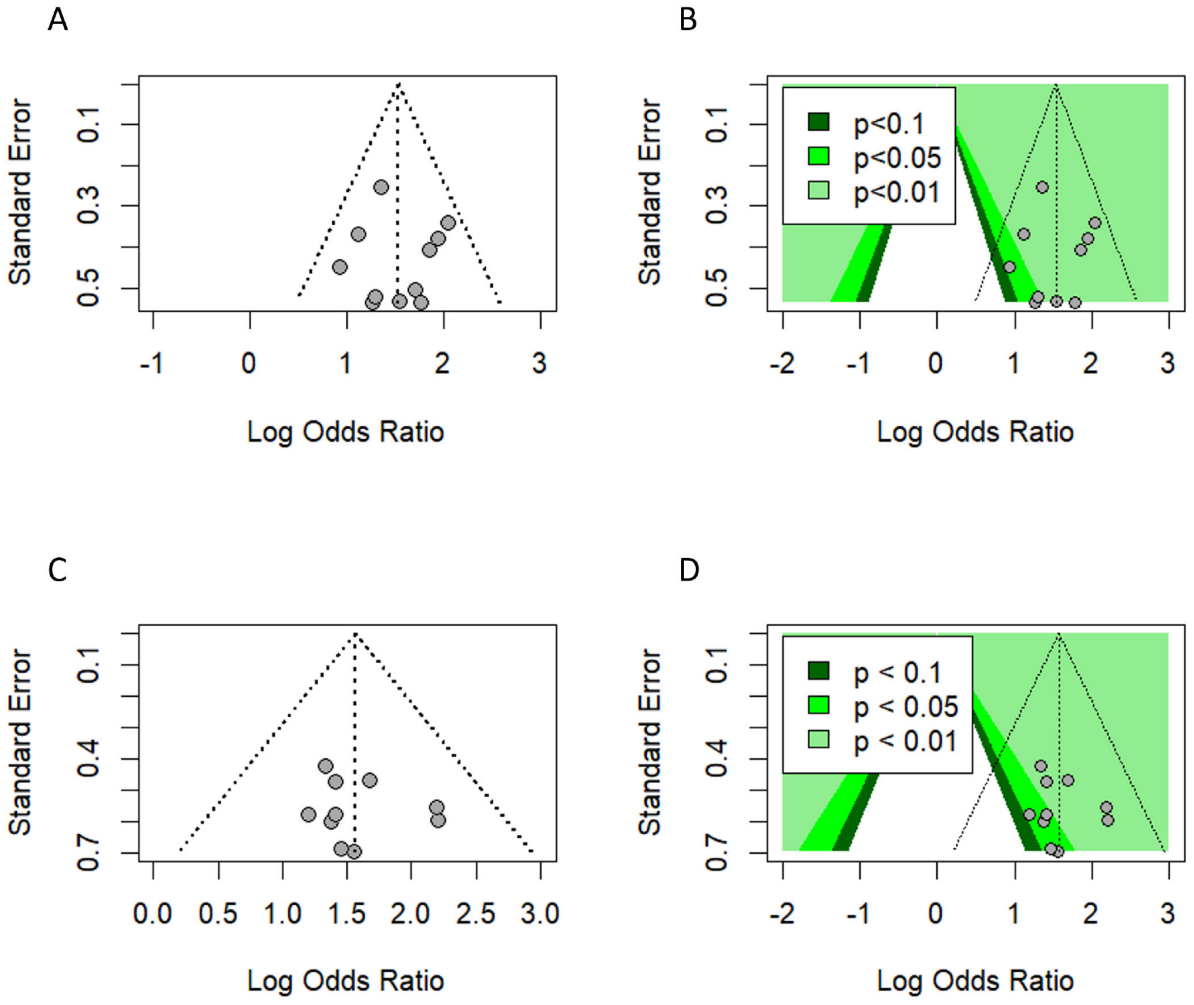
Publication bias. **(A, B)** Funnel plot and contour-enhanced funnel plot (univariate analysis). **(C, D)** Funnel plot and contour-enhanced funnel plot (multivariate analysis) with influential and outlier studies excluded.

We used a funnel plot to evaluate the presence of small study bias by visually inspecting the symmetry of the plot, imputing any missing effect estimate using the Duval and Tweedie trim-and-fill method and generating a contour-enhanced funnel plot (50, 51). After omitting influential studies, due to the limitations of the trim-and-fill method with the existence of high heterogeneity among studies (52), neither the visual inspection nor the trim-and-fill method showed any funnel plot asymmetry or missing effects, indicating no small study bias (**Figure 11**).

Additionally, Egger’s regression test did not suggest the presence of asymmetry in the funnel plot (53) (**Table 3**).

**Table 3:** Quantification of funnel plot asymmetry.

Study analysis	Test	Bias estimate	Confidence interval	t	P
Univariate	Egger’s regression test	0.022	-2.3-2.34	0.018	0.9858
Multivariate	Egger’s regression test	0.653	-1.91-3.21	0.500	0.6305

## 4 Discussion

The thoracolumbar fascia, also known as the lumbodorsal fascia, is an intricate multilayered connective tissue structure located in the posterior region of the trunk. Extending from the thoracic vertebrae to the sacrum, this fascia plays a vital role in maintaining the biomechanical stability of the spine and facilitating movements such as forward spinal flexion. Furthermore, it serves as an anchor point for various muscles along the vertebral column, enabling the distribution of forces across the trunk and enhancing core stability (20).

The precise mechanisms by which thoracolumbar fascia injury causes back pain remain unclear. However, based on the existing research, three mechanisms have been suggested for how TLFI contributes to back pain, and these proposed mechanisms may exist in isolation or in combination (16). First, by disrupting the structural integrity of the fascia, TLFI can cause micro-injuries or inflammation that may directly stimulate nociceptive nerve endings, which are specialized sensory nerve endings found in abundance within the fascial tissue. This direct stimulation can elicit back pain. A study conducted by Barry et al. (54) demonstrated that the thoracolumbar fascia contained approximately three times the concentration and distribution of sensory nerve fibers with calcitonin gene-related peptide (CGRP)-positive fibers compared with the back muscles. Similarly, Tesarz et al. (55) found that the fascia possesses a dense network of nociceptive nerves and the majority of CGRP-and substance P (SP)-containing sensory fibers are located in the outer layer of the fascia and subcutaneous tissue. Second, following TLF micro-injury and inflammation, restructuring, remodeling, or tissue stiffness is possible, leading to compromised functional integrity and proprioceptive signaling that alters the sensory input of fascial nociceptors (56). Finally, injury to the fascia can activate nociceptive nerve terminals, resulting in enhanced sensitivity and pain radiating from adjacent tissues with spinal connections similar to those of the thoracolumbar fascia (TLF) (16). Other studies have also indicated that these nerve endings proliferate following inflammatory or chemical stimulation in both experimental rats and humans, suggesting that fascial damage may contribute to back pain (57, 58). A study conducted by Schilder et al. (59) revealed the crucial role of the human thoracolumbar fascia in lower back pain. Their findings demonstrated that this tissue exhibits greater sensitivity to chemical stimuli than the muscle or subcutaneous tissues. This study involved artificially inducing inflammation by administering hypertonic saline into the thoracolumbar fascia, which elicited severe pain, extended pain duration, and a more extensive pain distribution pattern reminiscent of acute lower back pain symptoms. Moreover, the findings suggest that disruption or disorganization of fascial structures could be a contributing factor to chronic low back pain. These findings elucidate the role of the TLF as a significant etiological factor for back pain.

This systematic review and meta-analysis was conducted to assess the relationship between TLFI as an independent risk factor for the development of residual back pain following PVA treatment in patients with OVCFs. Our meta-analysis revealed that TLFI significantly increased the risk of RBP after PVA. The univariate analysis showed that patients with a TLFI were more than four times (OR: 4.62) more likely to develop postoperative pain than those without a TLFI, without adjusting for other related risk factors that may contribute to RBP. This result is consistent with previous findings (30). The sensitivity analysis indicated reduced heterogeneity to no heterogeneity after excluding two studies that contributed the majority of the observed heterogeneity, as shown in **Figures 7**, **9**. Similarly, after accounting for confounders and other related risk factors, the multivariate analysis confirmed an independent relationship between TLFI and the development of postoperative pain (OR: 4.79). This indicates that patients with a TLFI are more than four times more likely to develop residual back pain than those without a TLFI, demonstrating a strong association. Our findings are consistent with those of previous studies (33, 36). A sensitivity analysis was performed to ensure the robustness of the pooled effect estimate of the outcome and investigate the studies contributing to the heterogeneity observed in the analysis. After excluding three influential studies identified to be contributing to heterogeneity and affecting the overall effect estimate, there was no heterogeneity in the overall effect size, demonstrating the robustness of the overall effect estimate (**Figures 8**, **10**). Both the univariate and multivariate analyses robustly indicated that TLFI significantly increased the risk of RBP development after PVA. The sensitivity analysis confirmed that the observed associations were consistent and not unduly influenced by any individual study. The observed heterogeneity likely occurred due to statistical heterogeneity rather than methodological differences between the studies.

Recent studies have reported that osteoporotic vertebral compression fractures often involve thoracolumbar fascia injury, which is associated with residual back pain following percutaneous vertebral augmentation. A prospective cohort study by Yan et al. (60) investigated the causes of persistent back pain following vertebroplasty and found that fascia injury was present in 42.1% of the cases. Yang et al. (61) retrospectively analyzed the data of 132 patients with OVCFs and determined that TLFI has a substantial impact on the absence of immediate pain alleviation with a 39.4% incidence rate compared to those without a TLFI and it prolongs the ambulation time following PVP. Similarly, they reported that TLFI and associated pain could persist for over 3 months in certain patients, with follow-up MRI revealing worsened fascia injury compared to the preoperative condition, potentially contributing to prolonged postoperative pain. In this meta-analysis, the TLFI incidence rate was 28%, which indicates that fascia injury is a frequently overlooked complication that often coexists with osteoporotic vertebral fractures, underscoring the need for greater clinical attention. Osteoporotic vertebral compression fractures predominantly result from low-energy trauma and routine daily activities, such as lifting and twisting, or even occur without any noticeable trauma, rather than from high-impact trauma. This is due to the loss of bone quality and integrity (62, 63). However, this may not be the case for patients with OVCFs with a thoracolumbar fascia injury who are likely to have sustained high-impact trauma. A recent study by Deng et al. (17) evaluated the occurrence of TLFIs with an incidence of 27.8% in patients with OVCFs treated with PKP and found that the severity of fascia injury increased with the severity of trauma sustained by the patient. Moreover, they observed that a TLFI showed multilevel involvement, which was positively associated with the degree of trauma and impacted the efficacy of PKP, leading to acute residual pain. However, other medical situations in patients with OVCFs may contribute to fascia injury, which leads to postoperative pain following the augmentation procedure. In a retrospective study conducted by Luo et al. (18), TLFI contributed to RBP in patients with OVCFs after PVP. Simultaneously, they evaluated the risk factors that may lead to a TLFI and determined that a low body mass index (BMI), elevated blood pressure, and sarcopenia were significant risk factors for a TLFI. Sarcopenia was significantly more prevalent in the TLFI group than in the non-TLFI group, affecting 51.1% of individuals, compared to 14.7% in the non-TLFI cohort. Identifying these risk factors preoperatively can facilitate better risk stratification and counseling for patients undergoing PVA.

A TLFI is currently diagnosed by careful examination of the posterior fascia for any anomalous signals that may suggest the presence of edema on a preoperative MRI image. These signals may appear as low-intensity signals on T1WIs and as high-intensity signals on T2WIs and T2W1 fat-suppression sequences. Early detection and diagnosis of TLFI using advanced imaging modalities, such as MRI, can facilitate tailored interventions to alleviate residual pack pain. Thoracolumbar fascia injuries on MRI often present as elongated or flake-like patterns across multiple segments. These injuries are difficult to detect with only T1WIs and T2WIs but are clearly visible on T2WI fat-suppression imaging (17, 61). The development of standardized imaging protocols can ensure consistent detection and optimize preoperative planning. Although PVA procedures are effective treatment options for alleviating pain originating from OVCFs, they have no effect on improving the pain associated with fascia injury, which becomes obvious early in the postoperative period or the subsequent inflammation, and treating the fracture alone can result in suboptimal pain alleviation, patient dissatisfaction, and postoperative pain (61). Furthermore, reversing fascia injury may require a long recovery time. Langevin et al. (64) found that 4 weeks of passive stretching exercise did not restore fascia mobility following injury and removal of a movement restriction device, suggesting that a fascia injury could become a long-term issue and may not resolve automatically. Similarly, recent studies also suggest that TLFI and associated pain could persist longer in some individual patients and could even become exacerbated, leading to prolonged residual pain (61). These findings underscore the importance of identifying the origin of TLFI in patients with OVCFs to ensure successful PVA interventions. Additionally, considering patients with OVCFs are often elderly and have multiple comorbidities. The incidence of TLFI could be multifactorial and linked to natural age-related deterioration of the fascia due to overuse and poor posture. Furthermore, it could indicate severe traumatic injury or possibly suggest an undiagnosed comorbidity, such as sarcopenia, which is relatively common among elderly individuals with OVCFs (15, 17, 18, 31).

By recognizing TLFI as a risk factor for residual pain, it may be possible to devise more effective preoperative and postoperative patient care strategies and reduce the development of RBP. Preoperative treatment options may include focused pain management protocols such as localized anti-inflammatory injections to relieve TLFI-associated pain. Liu et al. (65) administered a cocktail of ropivacaine and betamethasone to a group of OVCF patients with TLFI prior to the PVP augmentation procedure. They compared the results to those of the control group and concluded that combined treatment can help alleviate pain, reduce the chance of RBP, and shorten the need for postoperative pain medication. However, this may prolong the duration of the surgery. Moreover, adding specific individually tailored physical therapy and functional exercises postoperatively aimed at helping recover thoracolumbar fascia integrity could help reduce fascia inflammation, mitigate chronic postoperative pain, and support fascia recovery (66, 67). Combining these preoperative medical interventions and postoperative physiotherapies based on individual patient needs may reduce postoperative pain associated with TLFI and its complications (61). Furthermore, although some degree of fascia damage is inevitable during vertebral augmentation procedures, reducing it as much as possible by more effectively utilizing navigation equipment such as C-arm fluoroscopy to better locate pedicles, reduce operation time, and reduce unnecessary needle punctures, particularly for OVCF patients with existing TLFI, could be an important step in mitigating persistent postoperative pain.

### 4.1 Implications of TLFI

The findings of this meta-analysis indicate that failing to address TLFI or its potential causes before and after surgery may prevent PVA treatment from fully achieving the intended outcomes of immediate pain alleviation and fracture stabilization. A thorough medical history and general health assessments of patients with OVCFs can aid physicians in differential diagnoses, and understanding the cause of the fracture traumatic or non-traumatic could be a vital step in ascertaining the origin of TLFI, as it eliminates trauma-related fascia injury. Currently, the presence of TLFI is reported in radiology reports as incidental soft tissue edema without any emphasis and many orthopedic doctors regard it as a minor issue, often attributing it to trauma, which is not applicable to all patients with OVCFs. Consequently, it is frequently overlooked compared to more pressing osteoporotic vertebral fractures. However, as demonstrated in this study, there is growing evidence indicating that a preoperative TLFI may have a negative clinical impact on the treatment outcome and pain relief in patients with OVCFs. A preoperative TLFI should be considered an indicator of early postoperative back pain (17). Additionally, considering that TLFI as a postoperative pain indicator goes beyond PVA, it could inform other musculoskeletal or spine-related conditions such as chronic back pain.

### 4.2 Strengths

This systematic review and meta-analysis has several strengths and limitations. To ensure the inclusion of all eligible studies, we conducted an exhaustive database search using a rigorous search strategy and criteria. We established a robust relationship between TLFI and postoperative residual pain through univariate and multivariate analyses, modifying the other risk factors in the multivariate analysis following PVA. In addition, we conducted a sensitivity analysis to investigate heterogeneity among the studies. After removing the two outliers that contributed to heterogeneity, we further confirmed the link between TLFI and RBP. Furthermore, this is the first meta-analysis to demonstrate an independent relationship between TLFI and RBP development after post-PVA treatment.

### 4.3 Limitations

In total, 12 of the 13 studies included in the analysis were retrospective cohort studies with a limited number of patients. These studies inherently contain bias and may introduce bias into the results of the review. Furthermore, some degree of variability may exist in the definition of fascia injury due to a lack of specific guidelines and definitions to follow, which may limit our conclusion. In addition, the data analyzed in this meta-analysis pertained to the short-term relationship between TLFI and RBP. There is a lack of information on the medium and long-term outcomes.

### 4.4 Future research

In order to validate the connection between TLFI and RBP after PVA, prospective studies are necessary. Researchers should also investigate the precise manner in which TLFI contributes to RBP, which could potentially mitigate this risk. Future studies should also evaluate the risk factors that may cause fascia injury in addition to the trauma associated with an OVCF, which may not always exist in patients, such as sarcopenia, which has been implicated in fascia injury. Current diagnostic procedures for what constitutes a TLFI and what degree of fascia injury should be considered a TLFI are lacking. To minimize this difference and the heterogeneity that inevitably arises from the lack of guidelines to follow, quantitative diagnostic methods are needed in future research.

## 5 Conclusion

This study demonstrated that preoperative TLFI is associated with postoperative residual pain after PVA, and the pooled effect consistently showed that, with or without the presence of other risk factors, patients with TLFIs have an increased risk of developing RBP. Recognizing fascia injury as a potential source of postoperative pain in clinical practice could enhance the care of these patients and mitigate postoperative pain. Additional research is needed to fully understand TLFIs and to develop effective treatments to reduce the risk of postoperative residual pain.

## Data Availability

The original contributions presented in the study are included in the article/**Supplementary Material**. Further inquiries can be directed to the corresponding author.

## References

[1] Johnell O Kanis JA . An estimate of the worldwide prevalence and disability associated with osteoporotic fractures. Osteoporos Int. (2006) 17:1726–33. doi: 10.1007/s00198-006-0172-4 16983459

[2] Sözen T Özışık L Başaran NÇ . An overview and management of osteoporosis. Eur J Rheumatol. (2017) 4:46–56. doi: 10.5152/eurjrheum.2016.048 28293453 PMC5335887

[3] Ong KL Beall DP Frohbergh M Lau E Hirsch JA . Were VCF patients at higher risk of mortality following the 2009 publication of the vertebroplasty “sham” trials? Osteoporos Int. (2018) 29:375–83. doi: 10.1007/s00198-017-4281-z PMC6394540 29063215

[4] Lau E Ong K Kurtz S Schmier J Edidin A . Mortality following the diagnosis of a vertebral compression fracture in the Medicare population. J Bone Joint Surg Am. (2008) 90:1479–86. doi: 10.2106/JBJS.G.00675 18594096

[5] Li F Eckstrom E Harmer P Fitzgerald K Voit J Cameron KA . Exercise and fall prevention: narrowing the research-to-practice gap and enhancing integration of clinical and community practice. J Am Geriatrics Soc. (2016) 64:425–31. doi: 10.1111/jgs.13925 PMC4760892 26825429

[6] Himič V Syrmos N Ligarotti GKI Kato S Fehlings MG Ganau M . The role of genetic and epigenetic factors in determining the risk of spinal fragility fractures: new insights in the management of spinal osteoporosis. Quantitative Imaging Med Surg. (2023) 13:7632645–7637645. doi: 10.21037/qims-23-513 PMC10644129 37969626

[7] Klazen CA Lohle PN de Vries J Jansen FH Tielbeek AV Blonk MC . Vertebroplasty versus conservative treatment in acute osteoporotic vertebral compression fractures (Vertos II): an open-label randomised trial. Lancet. (2010) 376:1085–92. doi: 10.1016/S0140-6736(10)60954-3 20701962

[8] Yang H Chen L Zheng Z Yin G Lu WW Wang G . Therapeutic effects analysis of percutaneous kyphoplasty for osteoporotic vertebral compression fractures: A multicentre study. J Orthopaedic Translation. (2017) 11:73–7. doi: 10.1016/j.jot.2017.04.003 PMC5866398 29662771

[9] Galibert P Deramond H Rosat P Le Gars D . Preliminary note on the treatment of vertebral angioma by percutaneous acrylic vertebroplasty. Neuro-chirurgie. (1987) 33:166–8. 3600949

[10] Yimin Y Zhiwei R Wei M Jha R . Current status of percutaneous vertebroplasty and percutaneous kyphoplasty – a review. Med Sci Monit. (2013) 19:826–36. doi: 10.12659/MSM.889479 PMC3795017 24097261

[11] Dohm M Black CM Dacre A Tillman JB Fueredi G . A randomized trial comparing balloon kyphoplasty and vertebroplasty for vertebral compression fractures due to osteoporosis. Am J Neuroradiology. (2014) 35:2227–36. doi: 10.3174/ajnr.A4127 PMC7965303 25300981

[12] Boonstra AM Schiphorst Preuper HR Balk GA Stewart RE . Cut-off points for mild, moderate, and severe pain on the visual analogue scale for pain in patients with chronic musculoskeletal pain. Pain. (2014) 155:2545–50. doi: 10.1016/j.pain.2014.09.014 25239073

[13] Gerbershagen HJ Rothaug J Kalkman CJ Meissner W . Determination of moderate-to-severe postoperative pain on the numeric rating scale: a cut-off point analysis applying four different methods. BJA: Br J Anaesthesia. (2011) 107:619–26. doi: 10.1093/bja/aer195 21724620

[14] Yang X-G Dong Y-Q Liu X Liu X-L Luo H-T Bao Y . Incidence and prognostic factors of residual back pain in patients treated for osteoporotic vertebral compression fractures: a systematic review and meta-analysis. Eur Spine J. (2024) 33:4521–37. doi: 10.1007/s00586-024-08426-z 39103616

[15] Peng Y Wu X Ma X Xu D Wang Y Xia D . Comparison between the clinical effect of percutaneous kyphoplasty for osteoporosis vertebral compression fracture patient with or without sarcopenia: A retrospective cohort study. IJGM. (2023) 16:3095–103. doi: 10.2147/IJGM.S423016 PMC10368018 37496597

[16] Wilke J Schleip R Klingler W Stecco C . The lumbodorsal fascia as a potential source of low back pain: A narrative review. BioMed Res Int. (2017) 2017:e5349620. doi: 10.1155/2017/5349620 PMC5444000 28584816

[17] Deng Z Feng T Wu X Xie H Song D Wang J . Thoracolumbar fascia injury in osteoporotic vertebral fracture: the important concomitant damage. BMC Musculoskeletal Disord. (2023) 24:166. doi: 10.1186/s12891-023-06280-6 PMC9987111 36879207

[18] Luo Y Jiang T Guo H Lv F Hu Y Zhang L . Osteoporotic vertebral compression fracture accompanied with thoracolumbar fascial injury: risk factors and the association with residual pain after percutaneous vertebroplasty. BMC Musculoskelet Disord. (2022) 23:343. doi: 10.1186/s12891-022-05308-7 35410277 PMC8996573

[19] Stecco C Macchi V Porzionato A Duparc F De Caro R . The fascia: the forgotten structure. IJAE: Ital J Anat Embryology. (2011) 116:127–38. doi: 10.1400/207563 22852442

[20] Willard FH Vleeming A Schuenke MD Danneels L Schleip R . The thoracolumbar fascia: anatomy, function and clinical considerations. J Anat. (2012) 221:507–36. doi: 10.1111/j.1469-7580.2012.01511.x PMC3512278 22630613

[21] Page MJ McKenzie JE Bossuyt PM Boutron I Hoffmann TC Mulrow CD . The PRISMA 2020 statement: an updated guideline for reporting systematic reviews. BMJ. (2021) 372:n71. doi: 10.1136/bmj.n71 33782057 PMC8005924

[22] Higgins JP Green S . Cochrane handbook for systematic reviews of interventions. Wiley. (2008). doi: 10.1002/9780470712184

[23] Yang J-S Liu J-J Chu L Li J Chen C Chen H . Causes of residual back pain at early stage after percutaneous vertebroplasty: A retrospective analysis of 1,316 cases. Pain Physician. (2019) 22:E495–503. doi: 10.36076/ppj/2019.22.e495 31561662

[24] Li Y Yue J Huang M Lin J Huang C Chen J . Risk factors for postoperative residual back pain after percutaneous kyphoplasty for osteoporotic vertebral compression fractures. Eur Spine J. (2020) 29:2568–75. doi: 10.1007/s00586-020-06493-6 32507918

[25] Li Q Shi L Wang Y Guan T Jiang X Guo D . A nomogram for predicting the residual back pain after percutaneous vertebroplasty for osteoporotic vertebral compression fractures. Pain Res Manage. (2021) 2021:1–12. doi: 10.1155/2021/3624614 PMC8575618 34760032

[26] Ge C Chen Z Lin Y Zheng Y Cao P Chen X . Preoperative prediction of residual back pain after vertebral augmentation for osteoporotic vertebral compression fractures: Initial application of a radiomics score based nomogram. Front Endocrinol. (2022) 13:1093508. doi: 10.3389/fendo.2022.1093508 PMC9816386 36619583

[27] Gao X Du J Hao D He B Yan L . Risk factors for residual back pain following percutaneous vertebral augmentation: the importance of paraspinal muscle fatty degeneration. Int Orthopaedics (SICOT). (2023) 47:1797–804. doi: 10.1007/s00264-023-05809-7 PMC10266997 37074374

[28] Lin M Wen X Huang Z Huang W Zhang H Huang X . A nomogram for predicting residual low back pain after percutaneous kyphoplasty in osteoporotic vertebral compression fractures. Osteoporos Int. (2023) 34:749–62. doi: 10.1007/s00198-023-06681-2 36738335

[29] Wang Z-W Wang G-Y Liu D-K Zhang D-Z Zhao C . Risk factors for residual back pain after PVP treatment for osteoporotic thoracolumbar compression fractures: A retrospective cohort study. World Neurosurg. (2023) 180:e484–93. doi: 10.1016/j.wneu.2023.09.094 37774786

[30] Wang R Xu Y Ma X . Risk factors and strategies for recovery quality, postoperative pain, and recurrent fractures between percutaneous kyphoplasty and percutaneous vertebroplasty in elderly patients with thoracolumbar compression fractures: a retrospective comparative cohort study. Ann Trans Med. (2023) 11:122–2. doi: 10.21037/atm-22-6475 PMC9929738 36819492

[31] Tu W Niu Y Su P Liu D Lin F Sun Y . Establishment of a risk prediction model for residual low back pain in thoracolumbar osteoporotic vertebral compression fractures after percutaneous kyphoplasty. J Orthop Surg Res. (2024) 19:41. doi: 10.1186/s13018-024-04528-y 38184651 PMC10771681

[32] Chen K Gao T Zhu Y Lyu F Jiang J Zheng C . Augmented Central Pain Processing Occurs after Osteoporotic Vertebral Compression Fractures and Is Associated with Residual Back Pain after Percutaneous Vertebroplasty. Asian Spine J. (2024) 18:380–9. doi: 10.31616/asj.2023.0429 PMC11222882 38764226

[33] Zhang Z . Risk factors for low back pain following percutaneous vertebroplasty in patients with osteoporotic vertebral compression fracture. Am J Transl Res. (2024) 16:3778–86. doi: 10.62347/SKKU1066 PMC11384397 39262739

[34] Shen L Yang H Zhou F Jiang T Jiang Z . Risk factors of short-term residual low back pain after PKP for the first thoracolumbar osteoporotic vertebral compression fracture. J Orthopaedic Surg Res. (2024) 19:792. doi: 10.1186/s13018-024-05295-6 PMC11590304 39587591

[35] Chen C Wu B Yu H Dai Z Yan L Cai D . Association between vertebral bone quality score and residual back pain following percutaneous vertebroplasty for osteoporotic vertebral compression fractures. Eur Spine J. (2024) 34:537–45. doi: 10.1007/s00586-024-08619-6 39688705

[36] Wells GA Shea B O’Connell D Peterson J Welch V Losos M . The Newcastle-Ottawa Scale (NOS) for assessing the quality of nonrandomised studies in meta-analyses. (2000).

[37] R Core Team . R: A Language and Environment for Statistical Computing (2023). Available online at: https://www.R-project.org/ (Accessed July 13, 2024).

[38] Balduzzi S Rücker G Schwarzer G . How to perform a meta-analysis with R: a practical tutorial. Evid Based Ment Health. (2019) 22:153–60. doi: 10.1136/ebmental-2019-300117 PMC10231495 31563865

[39] Harrer M Cuijpers P Furukawa T Ebert DD . dmetar: companion R package for the guide “Doing meta-analysis in R. (2019). doi: 10.5281/zenodo.2551802

[40] Harrer M Cuijpers P Furukawa TA Ebert DD . Doing Meta-Analysis with R: A Hands-On Guide. 1st ed. Boca Raton, FL and London: Chapman & Hall/CRC Press (2021).

[41] Lüdecke D . esc: effect size computation for meta analysis (Version 0.5.1). (2019). doi: 10.5281/zenodo.1249218

[42] DerSimonian R Laird N . Meta-analysis in clinical trials. Controlled Clin Trials. (1986) 7:177–88. doi: 10.1016/0197-2456(86)90046-2 3802833

[43] Knapp G Hartung J . Improved tests for a random effects meta-regression with a single covariate. Stat Med. (2003) 22:2693–710. doi: 10.1002/sim.1482 12939780

[44] Viechtbauer W Cheung MW-L . Outlier and influence diagnostics for meta-analysis. Res Synthesis Methods. (2010) 1:112–25. doi: 10.1002/jrsm.11 26061377

[45] Baujat B Mahé C Pignon J-P Hill C . A graphical method for exploring heterogeneity in meta-analyses: application to a meta-analysis of 65 trials. Stat Med. (2002) 21:2641–52. doi: 10.1002/sim.1221 12228882

[46] Olkin I Dahabreh IJ Trikalinos TA . GOSH - a graphical display of study heterogeneity. Res synthesis Methods. (2012) 3:214–23. doi: 10.1002/jrsm.1053 26062164

[47] Hartigan JA Wong MA . Algorithm AS 136: A K-means clustering algorithm. J R Stat Soc Ser C (Applied Statistics). (1979) 28:100–8. doi: 10.2307/2346830

[48] Schubert E Sander J Ester M Kriegel HP Xu X . DBSCAN revisited, revisited: why and how you should (Still) use DBSCAN. ACM Trans Database Syst. (2017) 42:1–21. doi: 10.1145/3068335

[49] Fraley C Raftery AE . Model-based clustering, discriminant analysis, and density estimation. J Am Stat Assoc. (2002) 97:611–31. doi: 10.1198/016214502760047131

[50] Duval S Tweedie R . Trim and fill: A simple funnel-plot–based method of testing and adjusting for publication bias in meta-analysis. Biometrics. (2000) 56:455–63. doi: 10.1111/j.0006-341X.2000.00455.x 10877304

[51] Peters JL Sutton AJ Jones DR Abrams KR Rushton L . Contour-enhanced meta-analysis funnel plots help distinguish publication bias from other causes of asymmetry. J Clin Epidemiol. (2008) 61:991–6. doi: 10.1016/j.jclinepi.2007.11.010 18538991

[52] Peters JL Sutton AJ Jones DR Abrams KR Rushton L . Performance of the trim and fill method in the presence of publication bias and between-study heterogeneity. Stat Med. (2007) 26:4544–62. doi: 10.1002/sim.2889 17476644

[53] Egger M Smith GD Schneider M Minder C . Bias in meta-analysis detected by a simple, graphical test. BMJ. (1997) 315:629–34. doi: 10.1136/bmj.315.7109.629 PMC2127453 9310563

[54] Barry CM Kestell G Gillan M Haberberger RV Gibbins IL . Sensory nerve fibers containing calcitonin gene-related peptide in gastrocnemius, latissimus dorsi and erector spinae muscles and thoracolumbar fascia in mice. Neuroscience. (2015) 291:106–17. doi: 10.1016/j.neuroscience.2015.01.062 25681518

[55] Tesarz J Hoheisel U Wiedenhöfer B Mense S . Sensory innervation of the thoracolumbar fascia in rats and humans. Neuroscience. (2011) 194:302–8. doi: 10.1016/j.neuroscience.2011.07.066 21839150

[56] Langevin HM Sherman KJ . Pathophysiological model for chronic low back pain integrating connective tissue and nervous system mechanisms. Med Hypotheses. (2007) 68:74–80. doi: 10.1016/j.mehy.2006.06.033 16919887

[57] Hoheisel U Rosner J Mense S . Innervation changes induced by inflammation of the rat thoracolumbar fascia. Neuroscience. (2015) 300:351–9. doi: 10.1016/j.neuroscience.2015.05.034 26003735

[58] Mense S . Innervation of the thoracolumbar fascia. Eur J Transl Myol. (2019) 29:8297. doi: 10.4081/ejtm.2019.8297 31579474 PMC6767935

[59] Schilder A Hoheisel U Magerl W Benrath J Klein T Treede R-D . Sensory findings after stimulation of the thoracolumbar fascia with hypertonic saline suggest its contribution to low back pain. Pain®. (2014) 155:222–31. doi: 10.1016/j.pain.2013.09.025 24076047

[60] Yan Y Xu R Zou T . Is thoracolumbar fascia injury the cause of residual back pain after percutaneous vertebroplasty? A prospective cohort study. Osteoporosis Int. (2015) 26:1119–24. doi: 10.1007/s00198-014-2972-2 25510580

[61] Yang S Tang J Yang Z Jin H Wang Q Wang H . Effect of thoracolumbar fascia injury on reported outcomes after percutaneous vertebroplasty. Front Surg. (2024) 11:1379769. doi: 10.3389/fsurg.2024.1379769 38817944 PMC11137208

[62] Yoo J-H Moon S-H Ha Y-C Lee DY Gong HS Park SY . Osteoporotic fracture: 2015 position statement of the Korean society for bone and mineral research. J Bone Metab. (2015) 22:175–81. doi: 10.11005/jbm.2015.22.4.175 PMC4691591 26713308

[63] Gerdhem P . Osteoporosis and fragility fractures: Vertebral fractures. Best Pract Res Clin Rheumatol. (2013) 27:743–55. doi: 10.1016/j.berh.2014.01.002 24836333

[64] Langevin HM Bishop J Maple R Badger GJ Fox JR . Effect of stretching on thoracolumbar fascia injury and movement restriction in a porcine model. Am J Phys Med Rehabil. (2018) 97:187. doi: 10.1097/PHM.0000000000000824 28901961 PMC7411307

[65] Liu X Zhou Q Sun Z Tian J Wang H . Clinical effects of cocktail injection on the thoracolumbar fascia injury during percutaneous vertebroplasty for osteoporotic vertebral compression fractures: a single-center, retrospective case-control study. BMC Musculoskelet Disord. (2024) 25:18. doi: 10.1186/s12891-023-07130-1 38166954 PMC10759409

[66] Berrueta L Muskaj I Olenich S Butler T Badger GJ Colas RA . Stretching impacts inflammation resolution in connective tissue. J Cell Physiol. (2016) 231:1621–7. doi: 10.1002/jcp.25263 PMC5222602 26588184

[67] Corey SM Vizzard MA Bouffard NA Badger GJ Langevin HM . Stretching of the back improves gait, mechanical sensitivity and connective tissue inflammation in a rodent model. PloS One. (2012) 7:e29831. doi: 10.1371/journal.pone.0029831 22238664 PMC3253101

