# Supplementary material for: Association between thoracolumbar fascia injury and residual back pain following percutaneous vertebral augmentation: a systematic review and meta-analysis
Source: Front Endocrinol (Lausanne). 2025 Apr 22;16:1532355. doi: 10.3389/fendo.2025.1532355 (PMC12052568; doi:10.3389/fendo.2025.1532355)
Supplement: Supplementary file 1 [file DataSheet1.docx]

# Search strategy

## PICOs Format

| P =***Population/Problem/Patients*** | Osteoporotic vertebral compression fracture |
| --- | --- |
| I = ***Intervention***/***Treatment*** | Vertebral Augmentation-VA (PVP, PKP) |
| C =***Comparison/Control*** | Non-Residual Back Pain Group |
| O= ***Outcome*** | Association of TLFI with Residual Back Pain |
| S= ***Study*** ***Design*** | Cohort studies OR randomized controlled trials |

## Mesh terms

| ***OVCFs*** | "Osteoporotic Fractures"[Mesh] OR "Spine"[Mesh] OR "Fractures, Compression"[Mesh] OR "Spinal Fractures"[Mesh] |
| --- | --- |
| ***VA*** | "Vertebroplasty"[Mesh] OR “Kyphoplasty"[Mesh] OR "Cementoplasty"[Mesh] OR "Bone Cements"[Mesh] |
| ***RBP*** | "Back Pain"[Mesh] OR “Low Back Pain"[Mesh] OR "Pain, Postoperative"[Mesh] OR "Chronic Pain"[Mesh] OR "Risk Factors"[Mesh] OR "Fascia"[Mesh] |

## ti, ab, kw-terms

| ***OVCFs*** | Osteoporotic vertebral compression fractures* OR OVCF* OR Osteoporotic thoracolumbar compression fractures* OR Osteoporotic vertebral fractures* |
| --- | --- |
| ***VA*** | Percutaneous vertebroplasty* OR percutaneous kyphoplasty* OR percutaneous cementoplasty* OR percutaneous vertebral augmentation* OR vertebral body augmentation* OR percutaneous spinal augmentation* OR vertebral augmentation* |
| ***RBP*** | Risk factor* OR Predictor* OR residual pain* OR residual back pain* OR residual low back pain* OR persistent back pain* OR chronic back pain* OR recurrent pain* OR thoracolumbar fascia injury* OR Chronic low back pain* |

## Combined Mesh terms and Ti, ab, kw terms into search Blocks

| ***#1*** | ***OVCFs*** | "Osteoporotic Fractures"[Mesh] OR "Spine"[Mesh] OR "Fractures, Compression"[Mesh] OR "Spinal Fractures"[Mesh] OR Osteoporotic vertebral compression fractures* OR OVCF* OR Osteoporotic thoracolumbar compression fractures* OR Osteoporotic vertebral fractures* |
| --- | --- | --- |
| ***#2*** | ***VA*** | "Vertebroplasty"[Mesh] OR Kyphoplasty "[Mesh] OR "Cementoplasty"[Mesh] OR "Bone Cements [Mesh] OR Percutaneous vertebroplasty* OR percutaneous kyphoplasty* OR percutaneous cementoplasty* OR percutaneous vertebral augmentation* OR vertebral body augmentation* OR percutaneous spinal augmentation* OR vertebral augmentation* |
| ***#3*** | ***RBP*** | "Back Pain"[Mesh] OR “Low Back Pain"[Mesh] OR "Pain, Postoperative"[Mesh] OR "Chronic Pain"[Mesh] OR "Fascia"[Mesh] OR "Risk Factors"[Mesh] OR Risk factors* OR Predictors* OR residual pain* OR residual back pain* OR residual low back pain* OR persistent pain* OR chronic back pain* OR recurrent pain* OR Chronic low back pain* OR thoracolumbar fascia injury* |
| ***#4*** | #1 AND #2 AND #3 | |

| Search | Number | Query | Results |
| --- | --- | --- | --- |
| “Exp OR ti, ab, kw” | #1 | ('fragility fracture')/exp OR ((spine)/exp) OR (('compression fracture')/exp) OR (('spine fracture')/exp) OR ('Osteoporotic vertebral compression fractures'):ti,ab,kw OR (('OVCF'):ti,ab,kw) OR (('Osteoporotic thoracolumbar compression fractures'):ti,ab,kw) OR (('Osteoporotic vertebral fracture'):ti,ab,kw) | 111,670 |
| “Exp OR ti, ab, kw” | #2 | ('Percutaneous vertebroplasty')/exp OR ((kyphoplasty)/exp) OR ((cementoplasty)/exp) OR (('bone cement')/exp) OR ('percutaneous vertebroplasty'):ti,ab,kw OR (('Percutaneous kyphoplasty'):ti,ab,kw) OR (('Percutaneous cementoplasty'):ti,ab,kw) OR (('Percutaneous vertebral augmentation'):ti,ab,kw) OR (('vertebral body augmentation'):ti,ab,kw) OR (('percutaneous spinal augmentation'):ti,ab,kw) OR (('vertebral augmentation'):ti,ab,kw) | 10,468 |
| “Exp OR ti, ab, kw” | #3 | (backache)/exp OR (('low back pain')/exp) OR (('postoperative pain')/exp) OR (('Chronic pain')/exp) OR (('risk factors')/exp) OR ((Fascia)/exp) OR ('risk factors'):ti,ab,kw OR ((predictors):ti,ab,kw) OR (('residual pain'):ti,ab,kw) OR (('residual back pain'):ti,ab,kw) OR (('residual low back pain'):ti,ab,kw) OR (('persistent back pain'):ti,ab,kw) OR (('Chronic back pain'):ti,ab,kw) OR (('recurrent pain'):ti,ab,kw) OR (( 'Chronic low back pain' ):ti,ab,kw) OR (('thoracolumbar fascia injury'):ti,ab,kw) | 1,067,256 |
|  | #4 | #1 AND #2 AND #3 | 1,421 |
|  | Filters applied: | AND [article]/lim AND [humans]/lim AND [english]/lim AND [embase]/lim AND [01-01-1966]/sd NOT [01-02-2024]/sd AND [<1966-2024]/py |  |

## Search results from ***Embase*** Database (***Search date:2024-12-31)***

| Search | Number | Query | Results |
| --- | --- | --- | --- |
| “All Fields” | #1 | ("Osteoporotic Fractures"[Mesh] OR "Spine"[Mesh] OR "Fractures, Compression"[Mesh] OR "Spinal Fractures"[Mesh] OR Osteoporotic vertebral compression fractures* OR OVCF* OR Osteoporotic thoracolumbar compression fractures* OR Osteoporotic vertebral fractures*) | 158,736 |
| “All Fields” | #2 | ("Vertebroplasty"[Mesh] OR Kyphoplasty "[Mesh] OR "Cementoplasty"[Mesh] OR "Bone Cements [Mesh] OR Percutaneous vertebroplasty* OR percutaneous kyphoplasty* OR percutaneous cementoplasty* OR percutaneous vertebral augmentation* OR vertebral body augmentation* OR percutaneous spinal augmentation* OR vertebral augmentation*) | 4,938 |
| “All Fields” | #3 | ("Back Pain"[Mesh] OR “Low Back Pain"[Mesh] OR "Pain, Postoperative"[Mesh] OR "Chronic Pain"[Mesh] OR "Fascia"[Mesh] OR "Risk Factors"[Mesh] OR Risk factors* OR Predictors* OR residual pain* OR residual back pain* OR residual low back pain* OR persistent pain* OR chronic back pain* OR recurrent pain* OR Chronic low back pain* OR thoracolumbar fascia injury*) | 2,087,116 |
|  | #4 | #1 AND #2 AND #3 (Filters applied =English) | 896 |

## Search results from ***PubMed*** Database (***Search date:2024-12-31, “ALL Field”***)

| Search | Number | Query | Results |
| --- | --- | --- | --- |
| “All Fields” | #1 | ("Osteoporotic Fractures"[Mesh] OR "Spine"[Mesh] OR "Fractures, Compression"[Mesh] OR "Spinal Fractures"[Mesh] OR Osteoporotic vertebral compression fractures* OR OVCF* OR Osteoporotic thoracolumbar compression fractures* OR Osteoporotic vertebral fractures*) | 157,590 |
| “All Fields” | #2 | ("Vertebroplasty"[Mesh] OR Kyphoplasty "[Mesh] OR "Cementoplasty"[Mesh] OR "Bone Cements [Mesh] OR Percutaneous vertebroplasty* OR percutaneous kyphoplasty* OR percutaneous cementoplasty* OR percutaneous vertebral augmentation* OR vertebral body augmentation* OR percutaneous spinal augmentation* OR vertebral augmentation*) | 3,934 |
| “All Fields” | #3 | ("Back Pain"[Mesh] OR “Low Back Pain"[Mesh] OR "Pain, Postoperative"[Mesh] OR "Chronic Pain"[Mesh] OR "Fascia"[Mesh] OR "Risk Factors"[Mesh] OR Risk factors* OR Predictors* OR residual pain* OR residual back pain* OR residual low back pain* OR persistent pain* OR chronic back pain* OR recurrent pain* OR Chronic low back pain* OR thoracolumbar fascia injury*) | 1,864,206 |
|  | #4 | #1 AND #2 AND #3 (Filters=English) | 808 |

## Search results from **MEDLINE** through PubMed Database (**search date:2024-12-31**)

| Search | Number | Query | Results |
| --- | --- | --- | --- |
| “All Fields” | #1 | ("Osteoporotic Fractures" OR "Spine" OR "Fractures, Compression" OR "Spinal Fractures" OR Osteoporotic vertebral compression fractures* OR OVCF* OR Osteoporotic thoracolumbar compression fractures* OR Osteoporotic vertebral fractures*) | 243,674 |
| “All Fields” | #2 | ("Vertebroplasty" OR Kyphoplasty " OR "Cementoplasty" OR "Bone Cements OR Percutaneous vertebroplasty* OR percutaneous kyphoplasty* OR percutaneous cementoplasty* OR percutaneous vertebral augmentation* OR vertebral body augmentation* OR percutaneous spinal augmentation* OR vertebral augmentation*) | 7,498 |
| “All Fields” | #3 | ("Back Pain" OR “Low Back Pain" OR "Pain, Postoperative" OR "Chronic Pain" OR "Fascia" OR "Risk Factors" OR Risk factors* OR Predictors* OR residual pain* OR residual back pain* OR residual low back pain* OR persistent pain* OR chronic back pain* OR recurrent pain* OR Chronic low back pain* OR thoracolumbar fascia injury*) | 2,059,752 |
|  | #4 | #1 AND #2 AND #3 (Filters applied= Articles and English) | 1,176 |

## Search results from Web of science Core Database (Search date:2024-12-31)

| Search | Number | Query | Results |
| --- | --- | --- | --- |
| “Mesh descriptor/Exp all trees” | #1 | [Osteoporotic Fractures" OR "Spine" OR "Fractures, Compression" OR "Spinal Fractures] | 8,262 |
| “ti, ab, kw/word variations searched” | #2 | (Osteoporotic vertebral compression fractures* OR OVCF* OR Osteoporotic thoracolumbar compression fractures* OR Osteoporotic vertebral fractures*) | 1,277 |
| “Combined, tiab, kw, Mesh descriptors” | #3 | #1 AND #2 | 567 |
| “Mesh descriptor/Exp all trees” | #4 | [Vertebroplasty" OR Kyphoplasty " OR "Cementoplasty" OR "Bone Cements] | 756 |
| “ti, ab, kw/word variations searched” | #5 | (Percutaneous vertebroplasty* OR percutaneous kyphoplasty* OR percutaneous cementoplasty* OR percutaneous vertebral augmentation* OR vertebral body augmentation* OR percutaneous spinal augmentation* OR vertebral augmentation*) | 592 |
| “Combined, tiab, kw, Mesh descriptors” | #6 | #4 AND #5 | 238 |
| “Mesh descriptor/Exp all trees” | #7 | [Back Pain" OR “Low Back Pain" OR "Pain, Postoperative" OR "Chronic Pain" OR "Fascia" OR "Risk Factors] | 72,282 |
| “ti, ab, kw/word variations searched” | #8 | (Risk factors* OR Predictors* OR residual pain* OR residual back pain* OR residual low back pain* OR persistent pain* OR chronic back pain* OR recurrent pain* OR Chronic low back pain* OR thoracolumbar fascia injury*) | 189,419 |
| “Combined, tiab, kw, Mesh descriptors” | #9 | #7 AND #8 | 45,602 |
|  | #10 | #3 AND #6 AND #9 | 15 |

## Search results from Cochrane library central Database (Search date:2024-12-31)
