# Supplementary material for: Association between thoracolumbar fascia injury and residual back pain following percutaneous vertebral augmentation: a systematic review and meta-analysis
Source: Front Endocrinol (Lausanne). 2025 Apr 22;16:1532355. doi: 10.3389/fendo.2025.1532355 (PMC12052568; doi:10.3389/fendo.2025.1532355)
Supplement: Supplementary file 2 [file DataSheet2.docx]

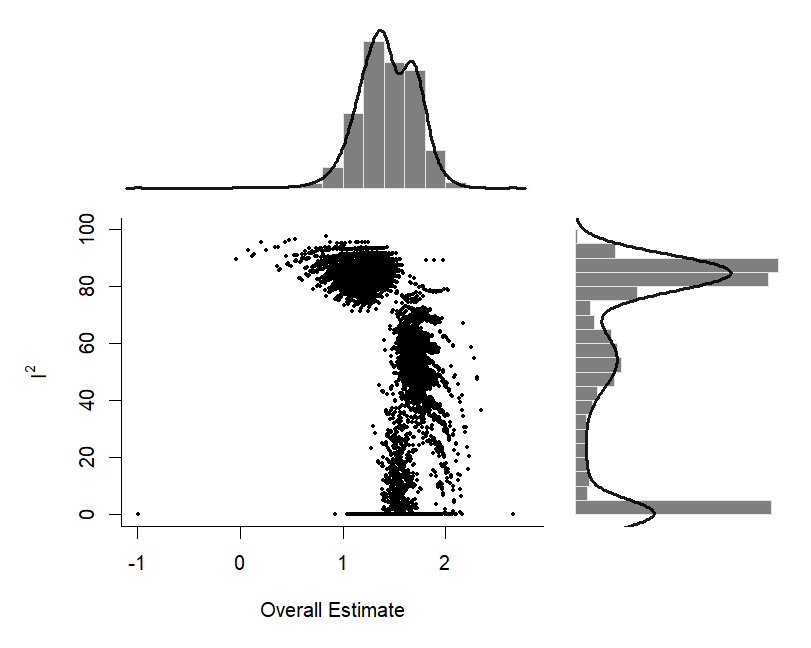


**Figure 1:** Gosh-plot sensitivity analysis demonstrating high-heterogeneity and high effect size -Univariate analysis result.


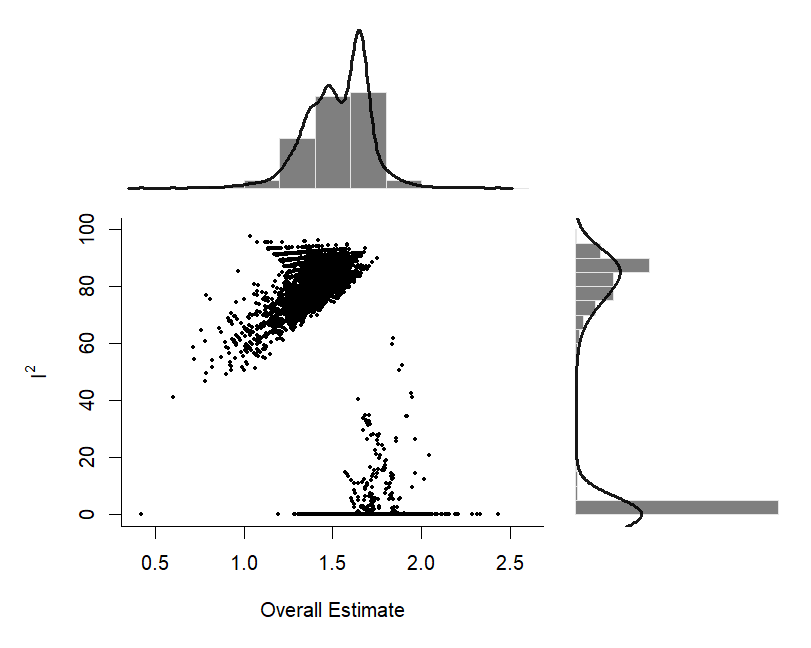


**Figure 2 :** Gosh-plot sensitivity analysis demonstrating high-heterogeneity and high effect size -Multivariate analysis result.
